# Supplementary figures and images for: Executioner caspase is proximal to Fasciclin 3 which facilitates non-lethal activation in Drosophila olfactory receptor neurons
Source: eLife. 2025 Jun 17;13:RP99650. doi: 10.7554/eLife.99650 (PMC12173457; doi:10.7554/eLife.99650)

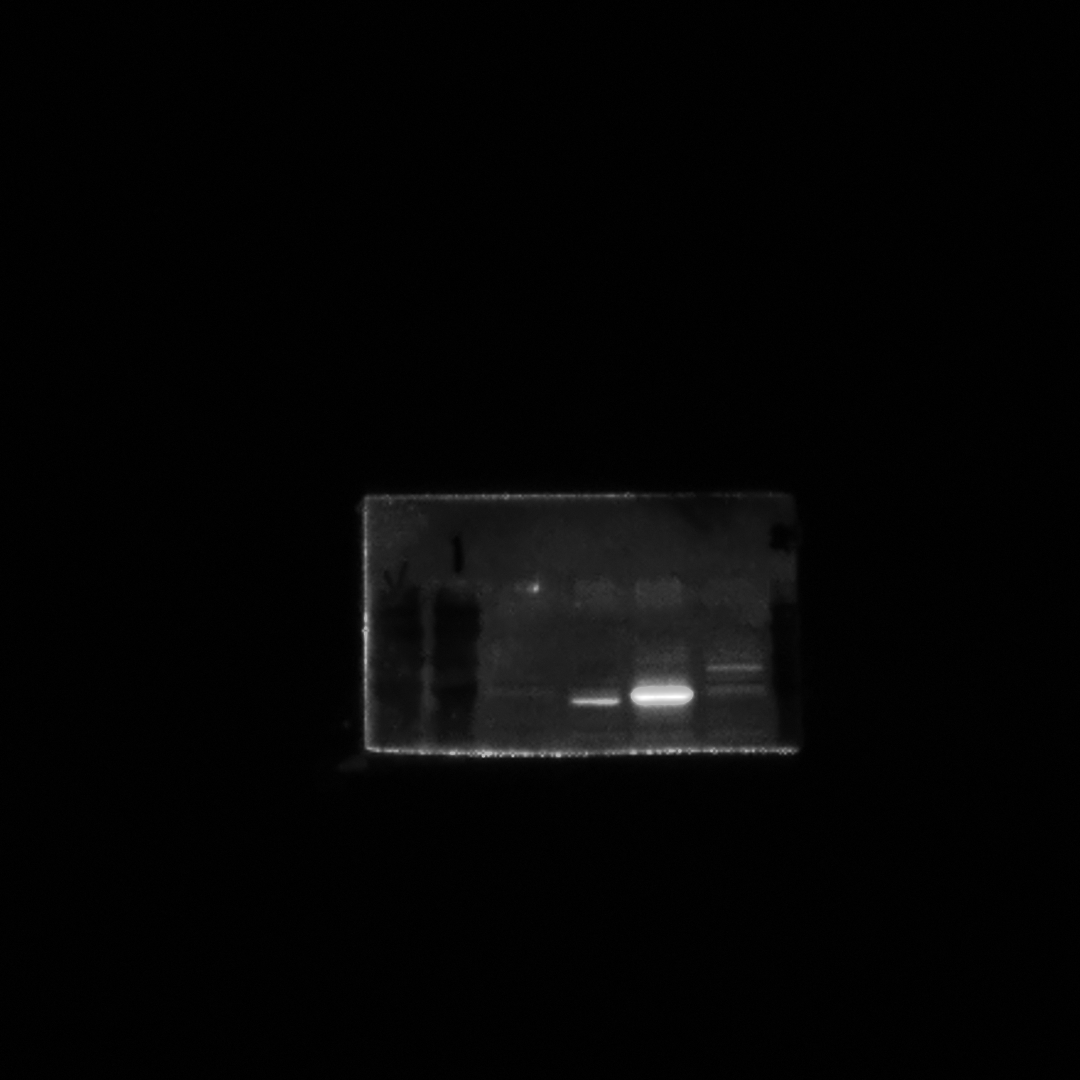

Supplement: Figure 1—source data 1. [file elife-99650-fig1-data1.zip › Figure 1 - Source Data 1/Figure1B_V5_raw.Tif]

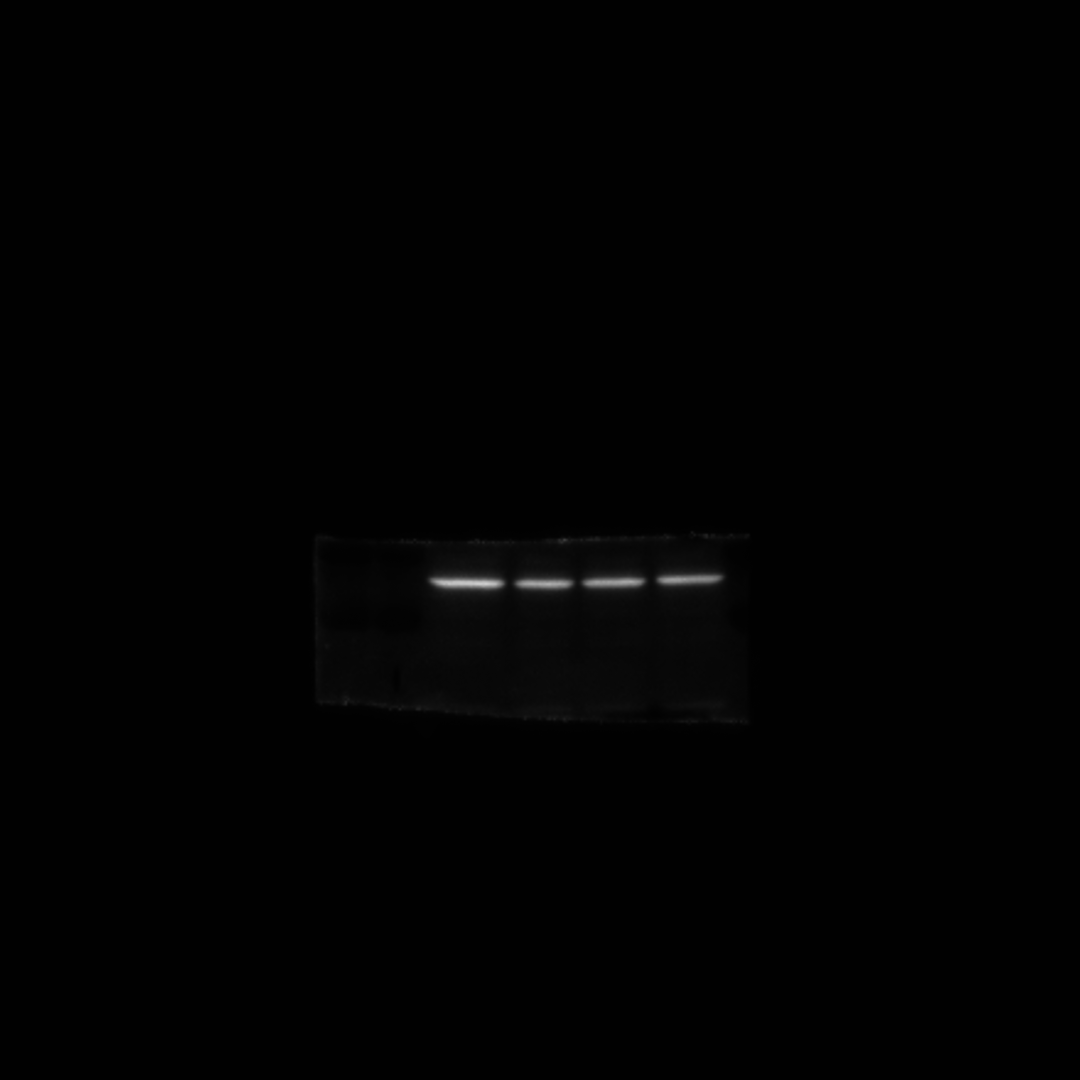

Supplement: Figure 1—source data 1. [file elife-99650-fig1-data1.zip › Figure 1 - Source Data 1/Figure1B_Actin_raw.Tif]

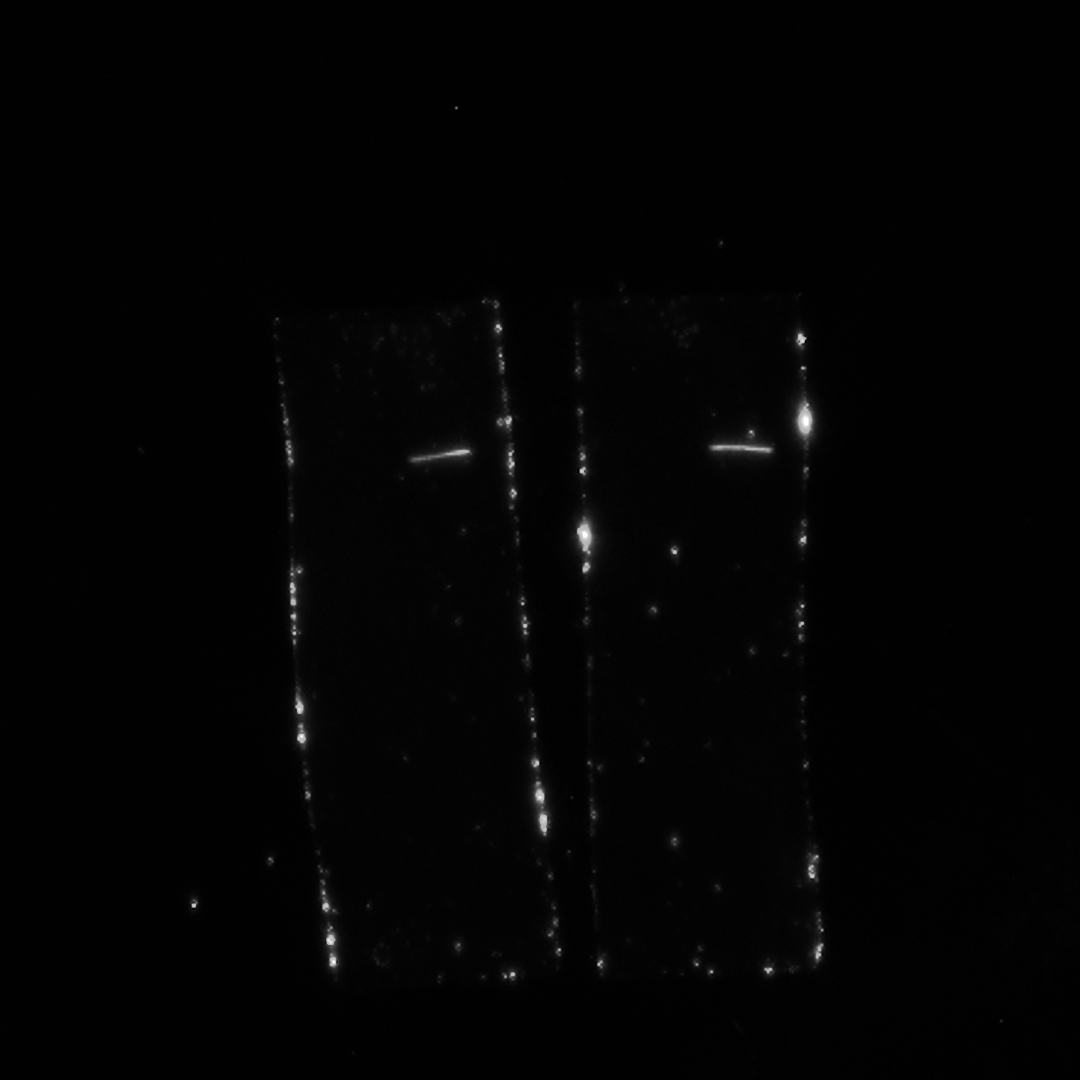

Supplement: Figure 1—source data 1. [file elife-99650-fig1-data1.zip › Figure 1 - Source Data 1/Figure1E_V5_raw.Tif]

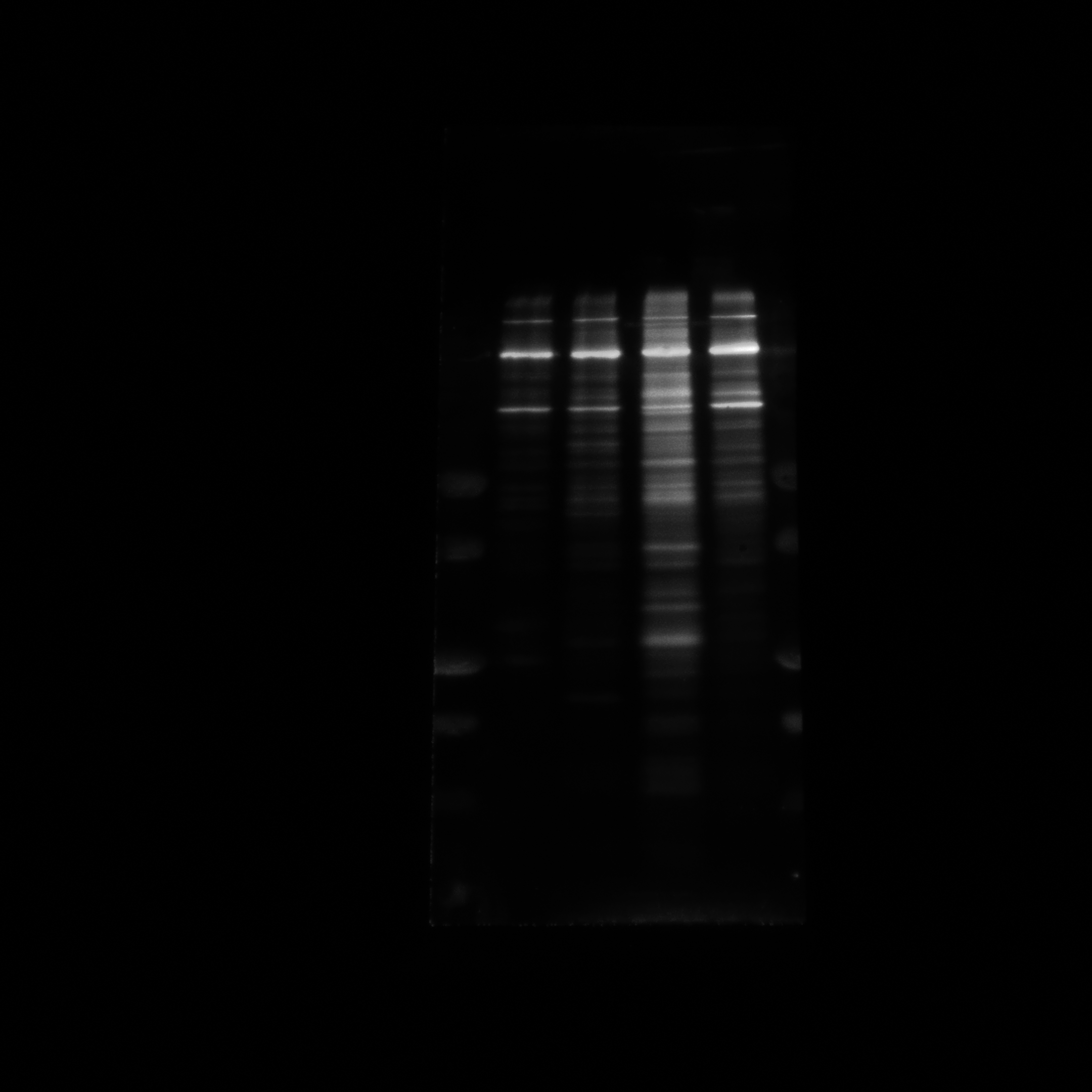

Supplement: Figure 1—source data 1. [file elife-99650-fig1-data1.zip › Figure 1 - Source Data 1/Figure1B_Streptavidin_raw.Tif]

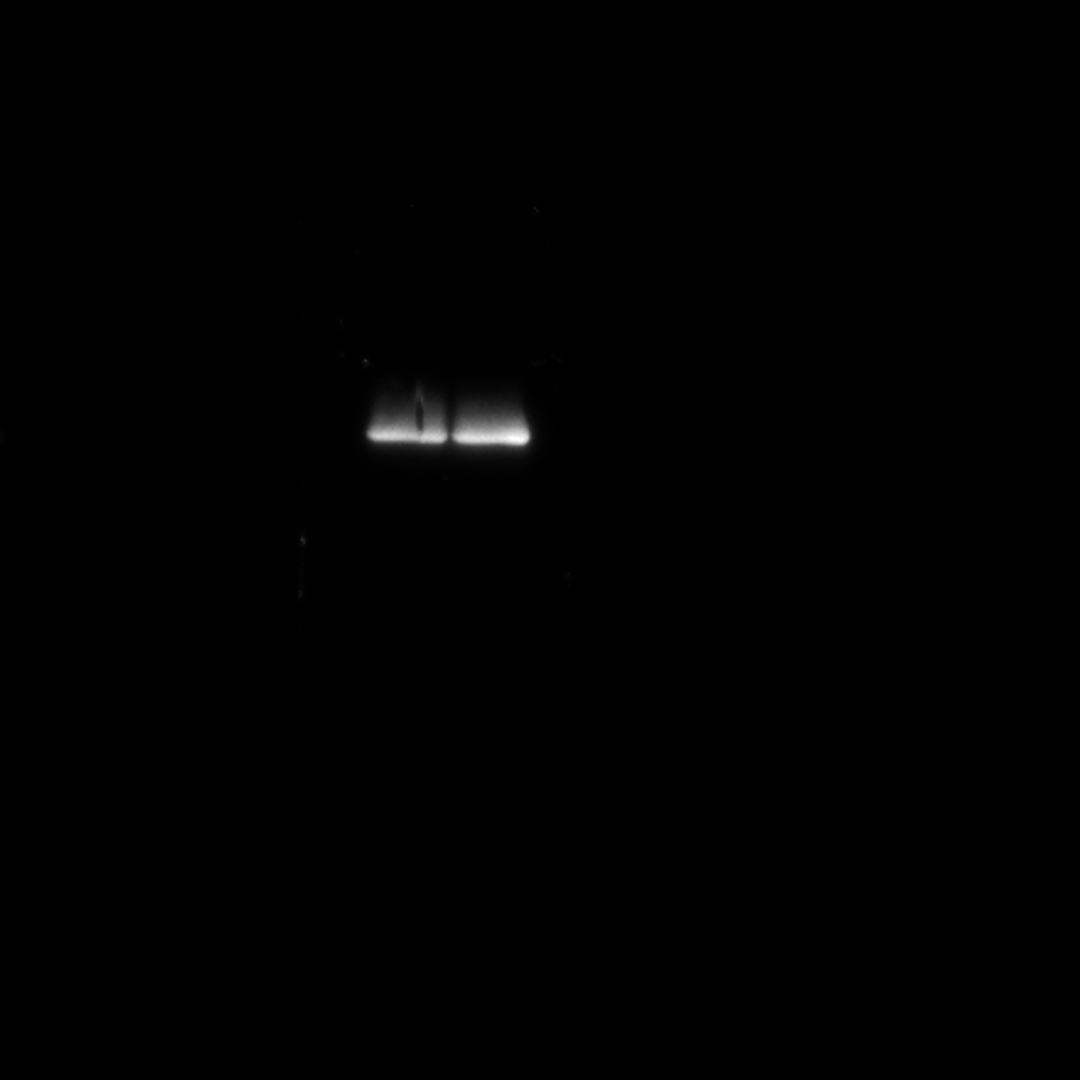

Supplement: Figure 1—source data 1. [file elife-99650-fig1-data1.zip › Figure 1 - Source Data 1/Figure1E_Tubulin_raw.Tif]

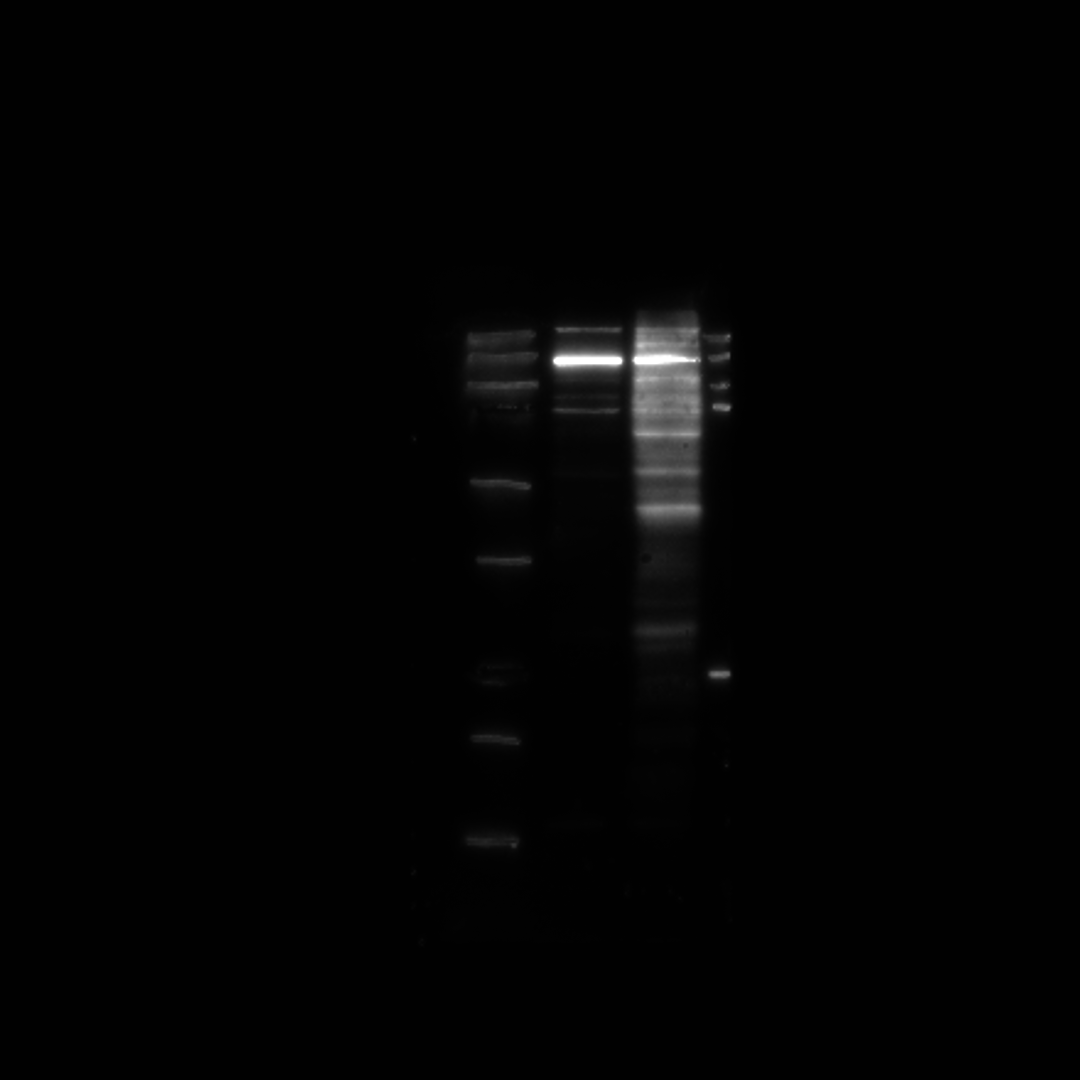

Supplement: Figure 1—source data 1. [file elife-99650-fig1-data1.zip › Figure 1 - Source Data 1/Figure1E_Streptavidin_raw.Tif]

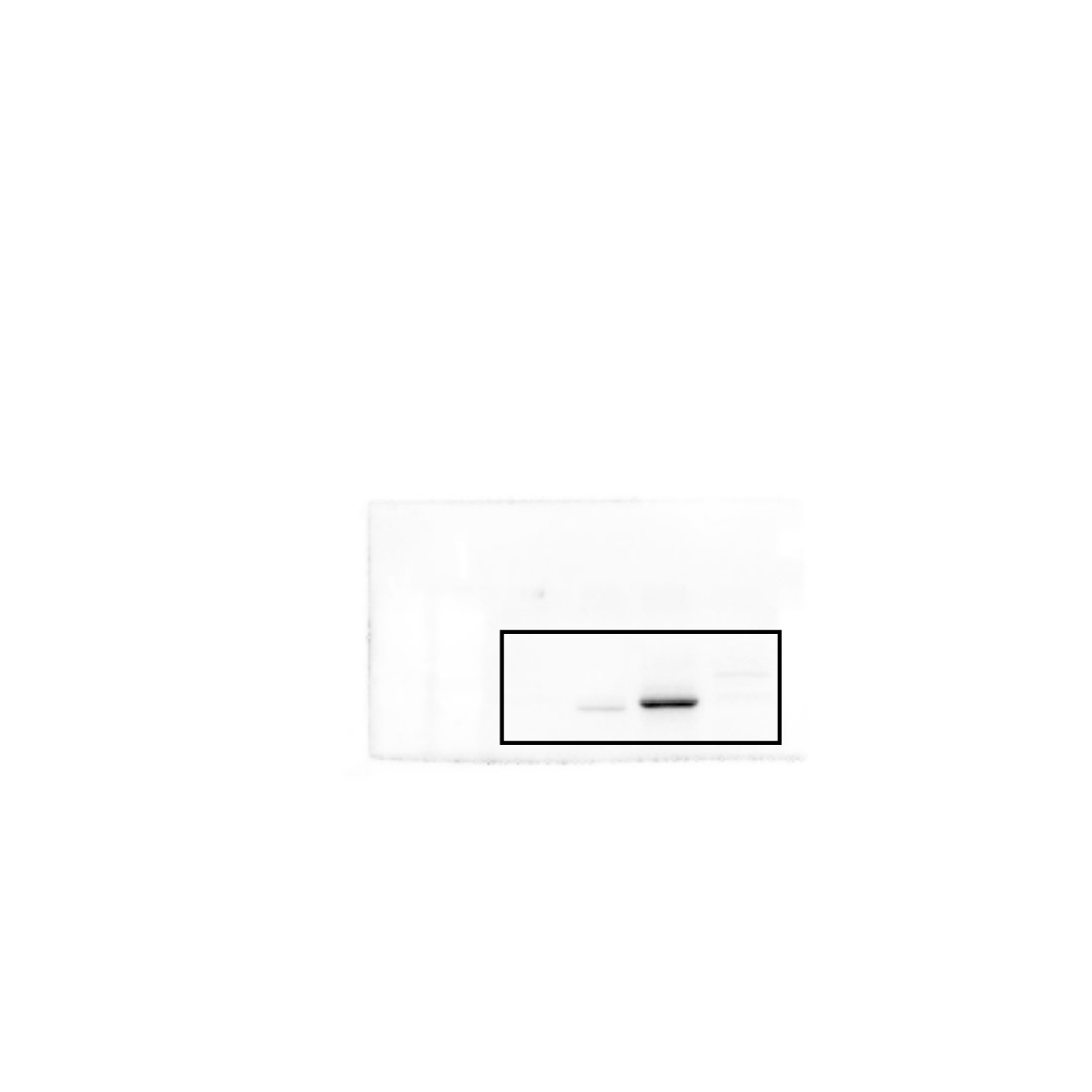

Supplement: Figure 1—source data 2. [file elife-99650-fig1-data2.zip › Figure 1 - Source Data 2/Figure1B_V5_annotated.tif]

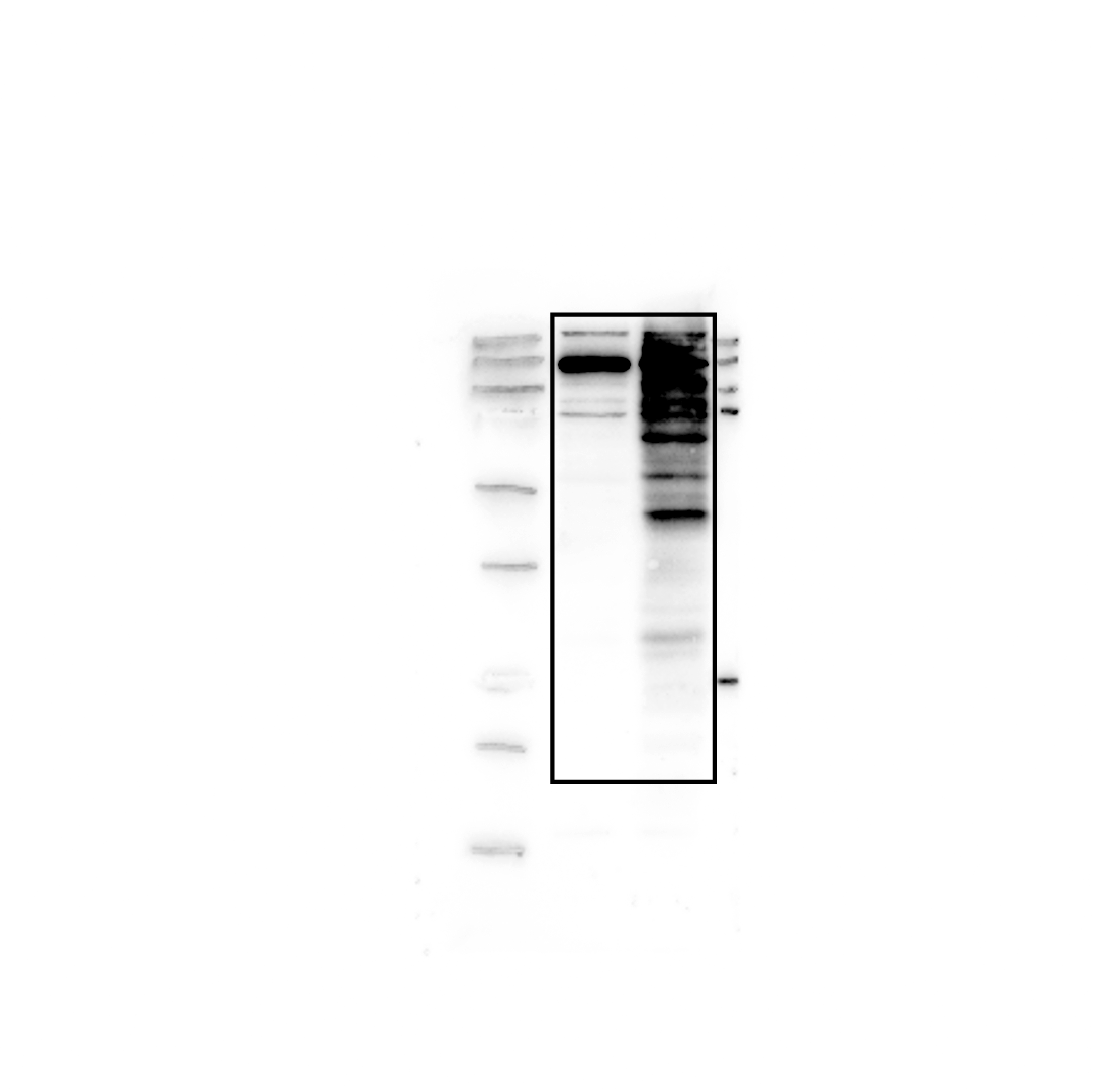

Supplement: Figure 1—source data 2. [file elife-99650-fig1-data2.zip › Figure 1 - Source Data 2/Figure1E_Streptavidin_annotated.tif]

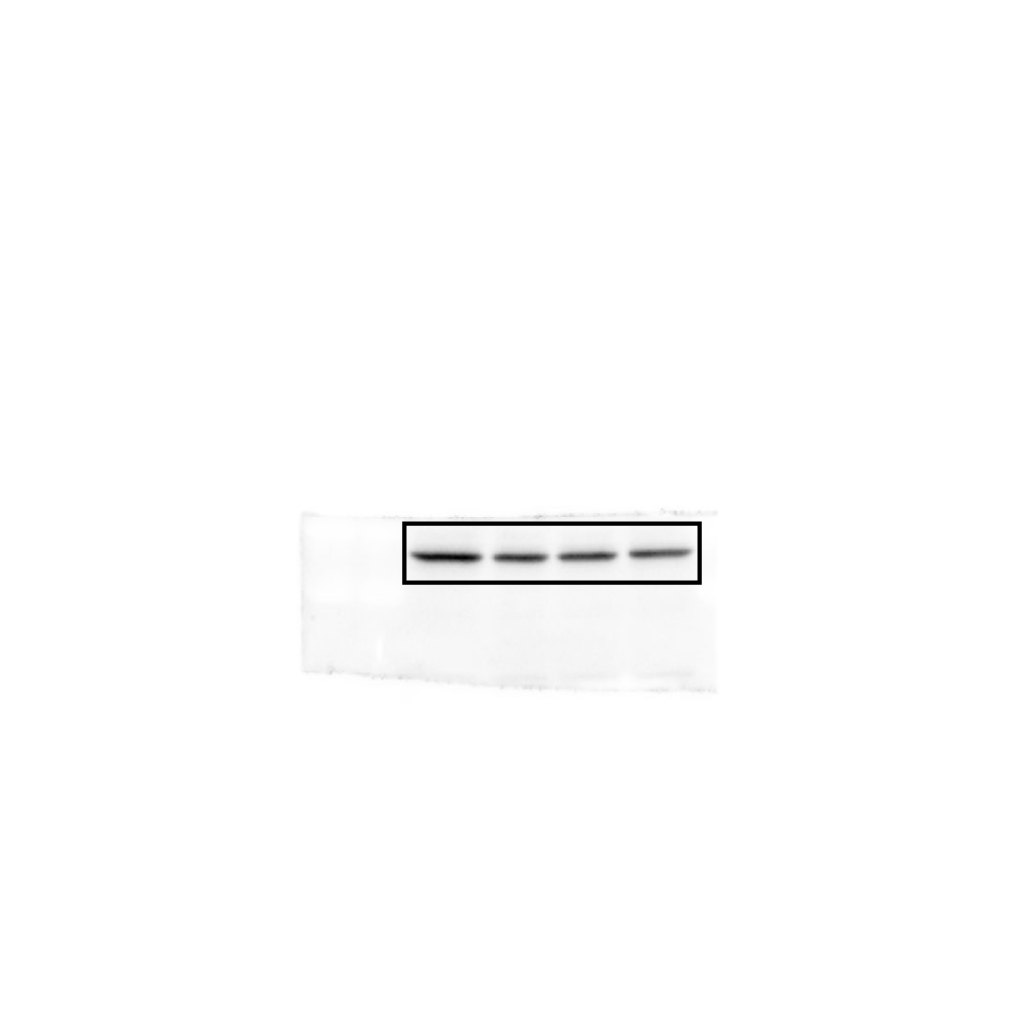

Supplement: Figure 1—source data 2. [file elife-99650-fig1-data2.zip › Figure 1 - Source Data 2/Figure1B_Actin_annotated.tif]

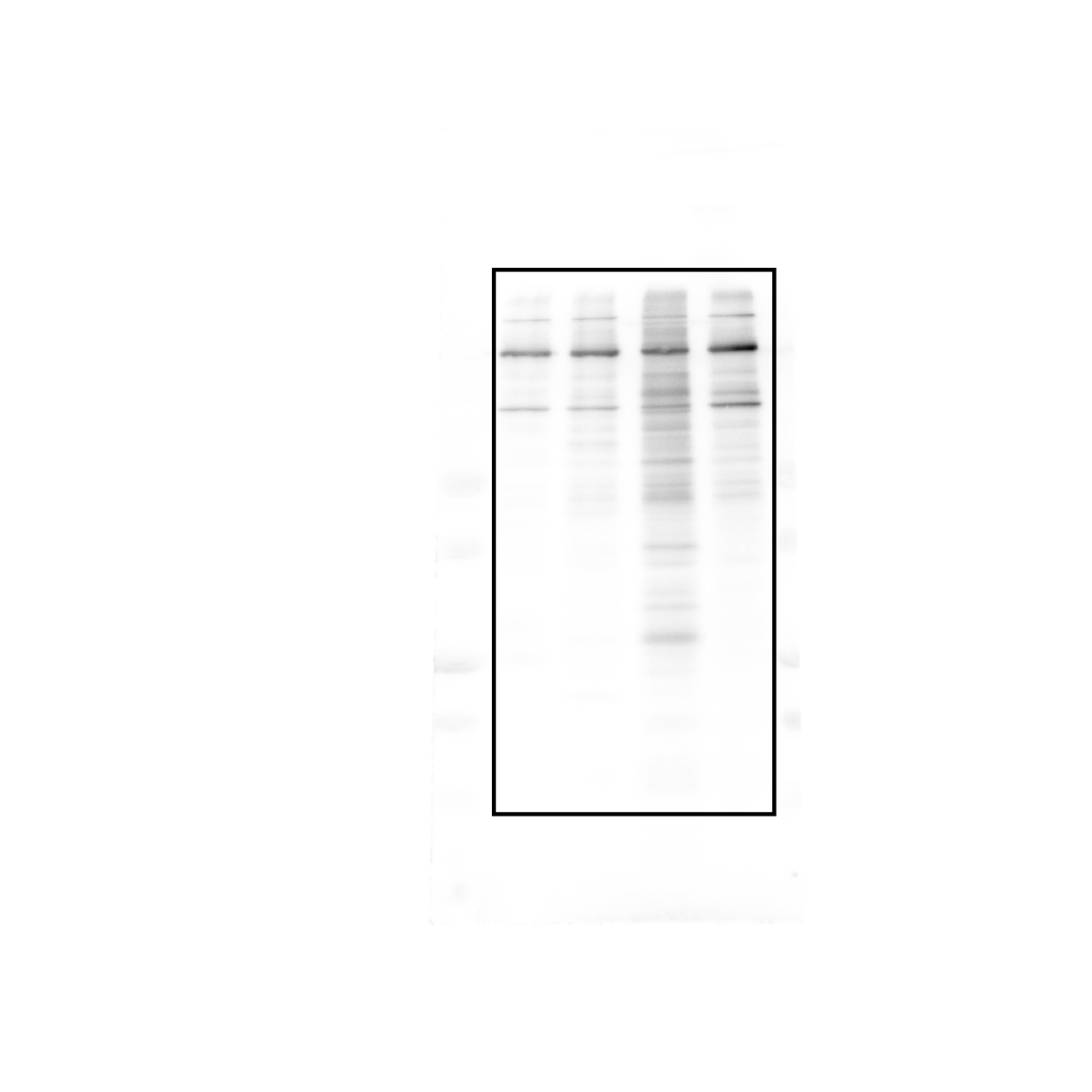

Supplement: Figure 1—source data 2. [file elife-99650-fig1-data2.zip › Figure 1 - Source Data 2/Figure1B_Streptavidin_annotated.tif]

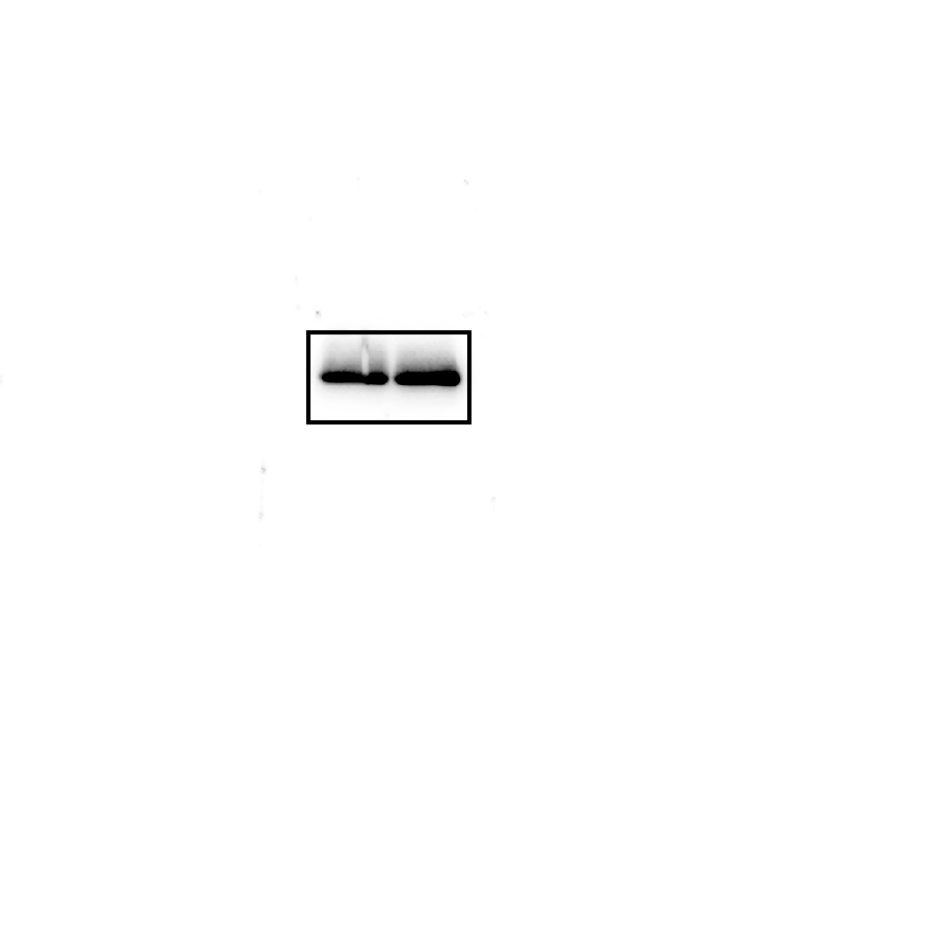

Supplement: Figure 1—source data 2. [file elife-99650-fig1-data2.zip › Figure 1 - Source Data 2/Figure1E_Tubulin_annotated.tif]

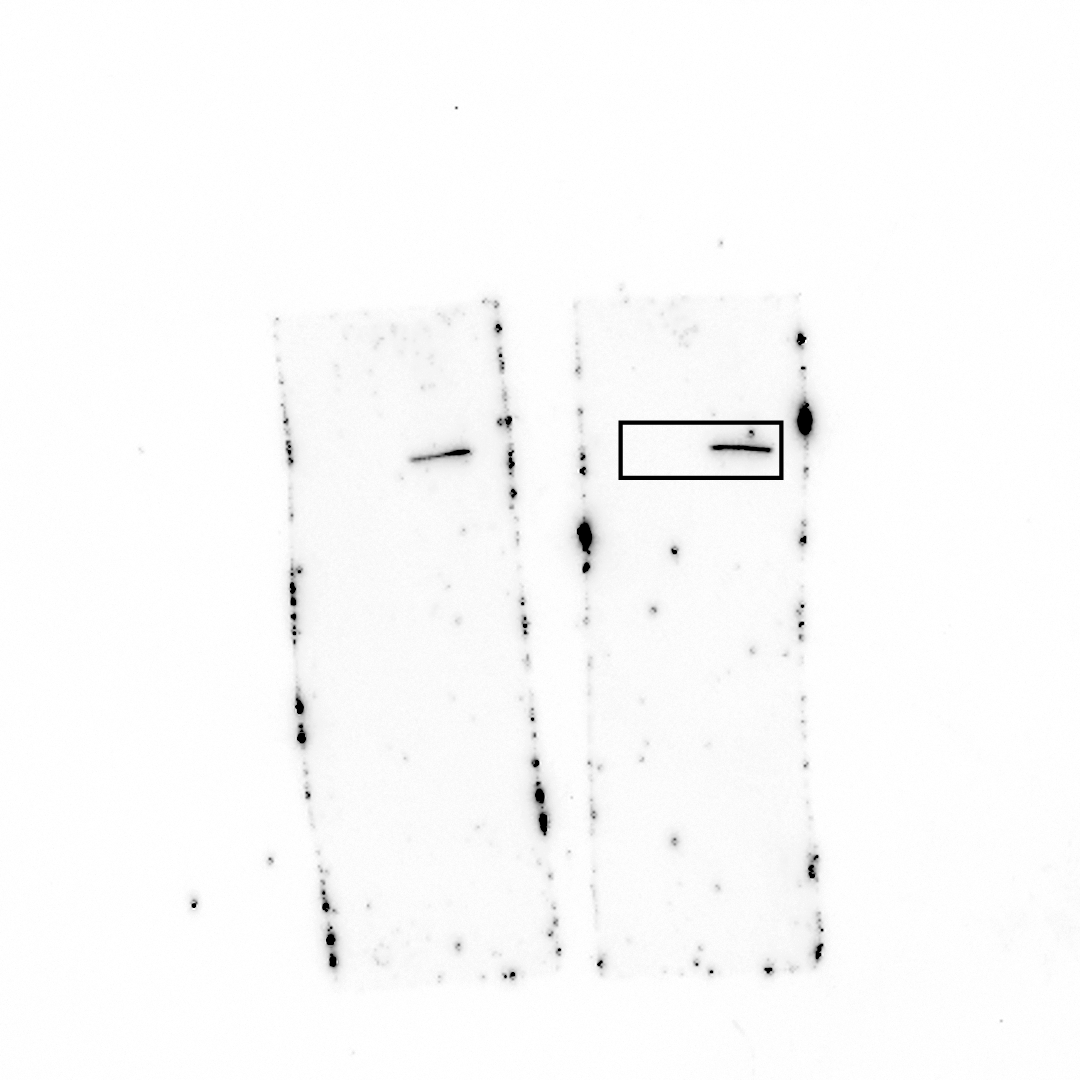

Supplement: Figure 1—source data 2. [file elife-99650-fig1-data2.zip › Figure 1 - Source Data 2/Figure1E_V5_annotated.tif]

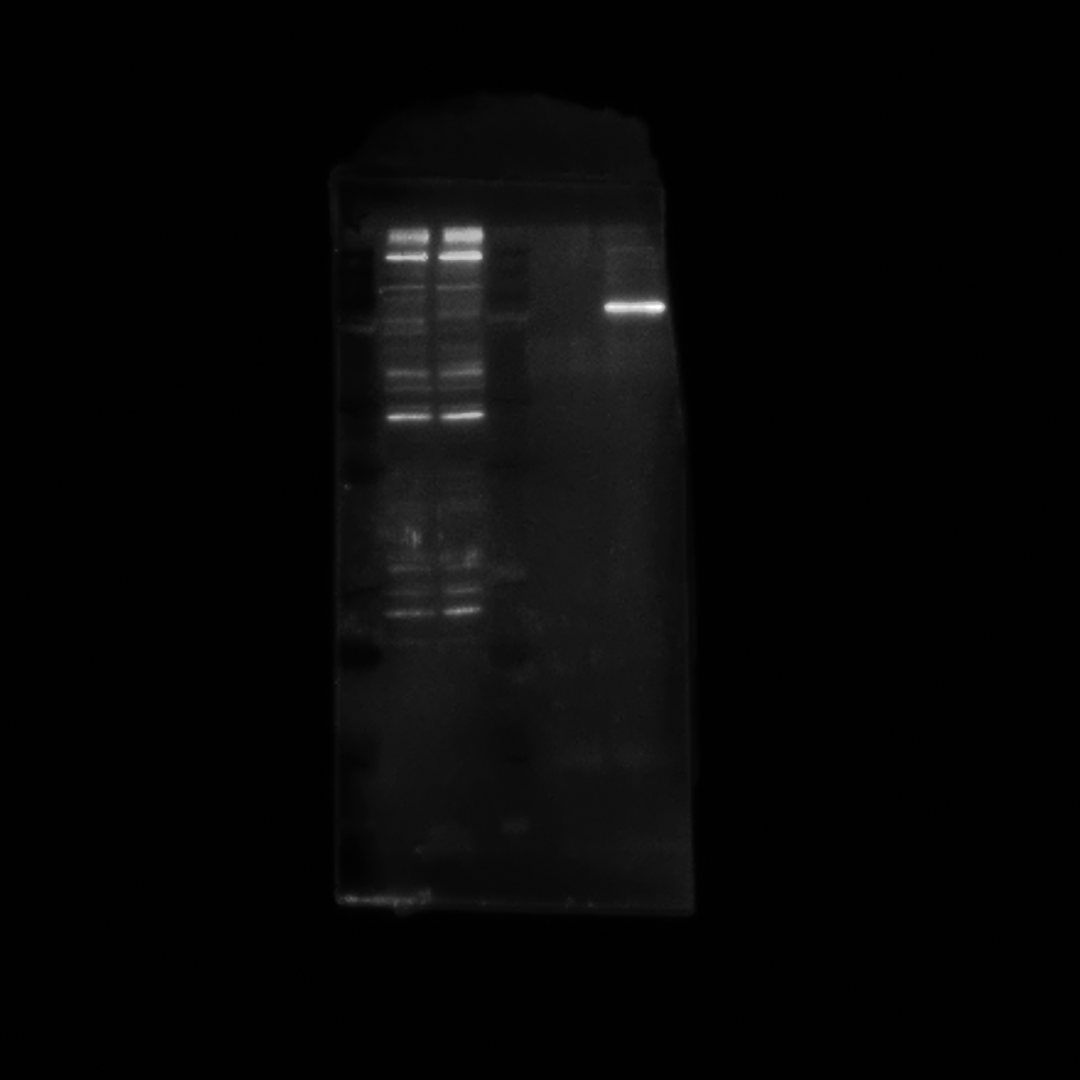

Supplement: Figure 2—source data 1. [file elife-99650-fig2-data1.zip › Figure 2 - Source Data 1/Figure2F_Fas3G_raw.Tif]

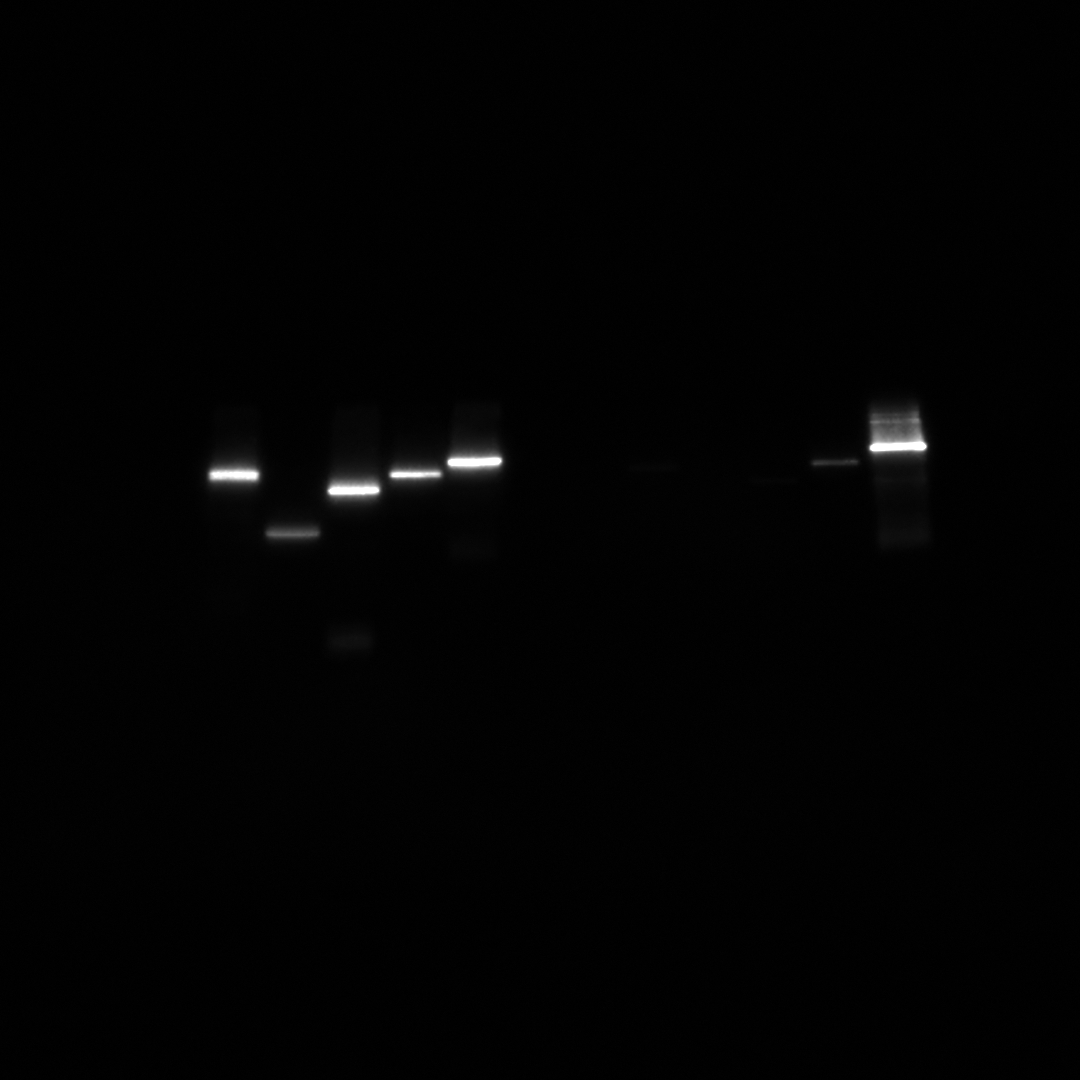

Supplement: Figure 2—source data 1. [file elife-99650-fig2-data1.zip › Figure 2 - Source Data 1/Figure2G_FLAG_raw.Tif]

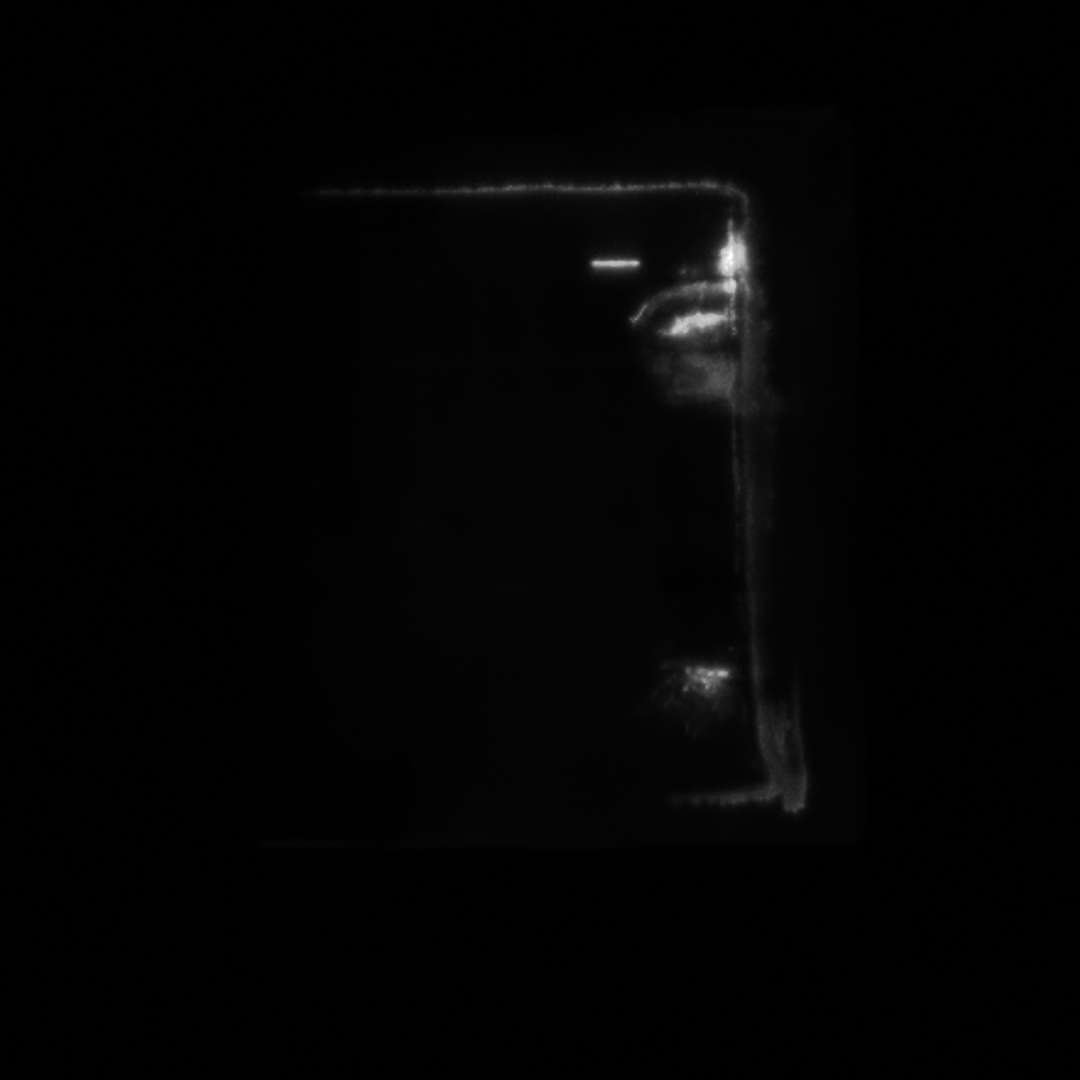

Supplement: Figure 2—source data 1. [file elife-99650-fig2-data1.zip › Figure 2 - Source Data 1/Figure2E_Fas3G_raw.Tif]

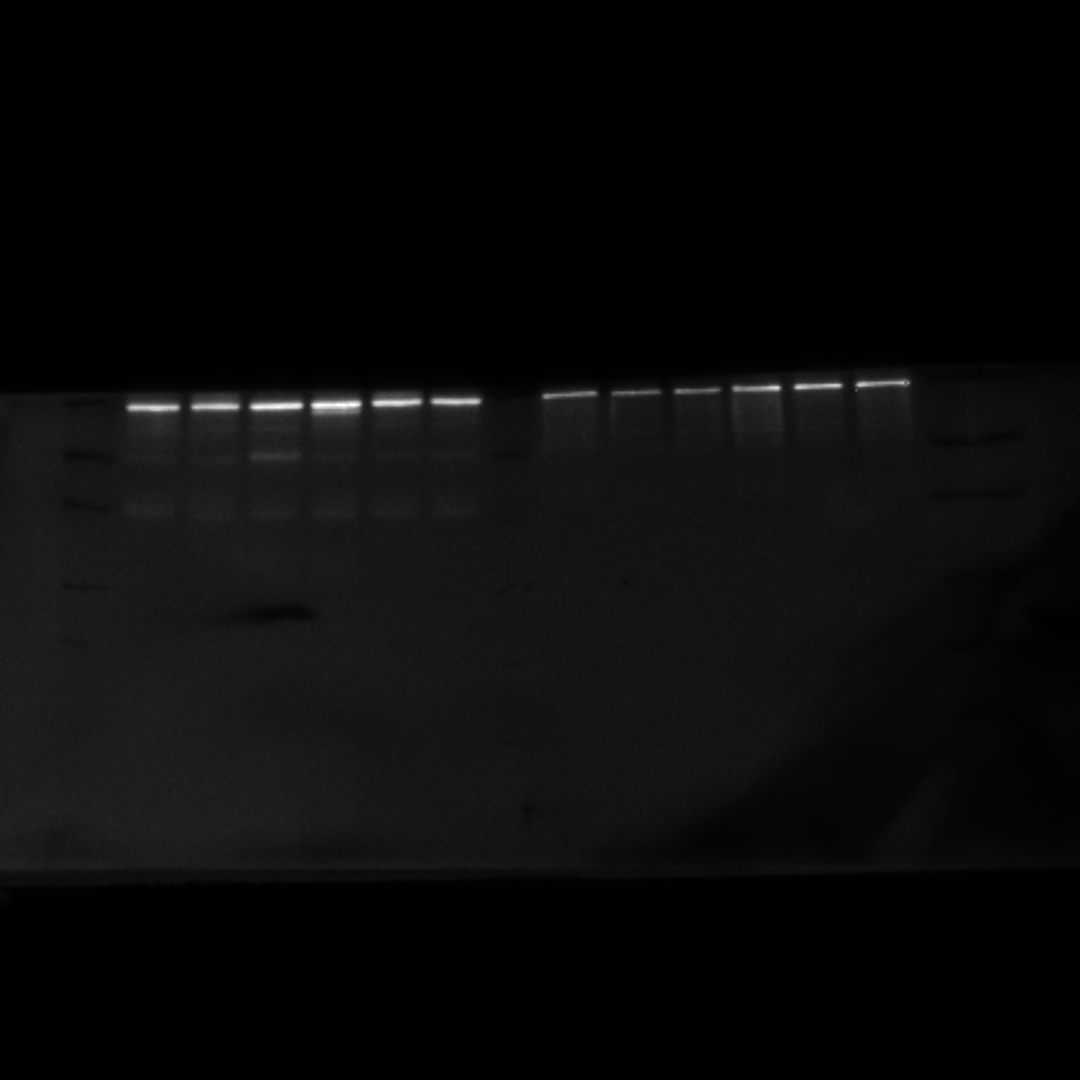

Supplement: Figure 2—source data 1. [file elife-99650-fig2-data1.zip › Figure 2 - Source Data 1/Figure2G_V5_raw.Tif]

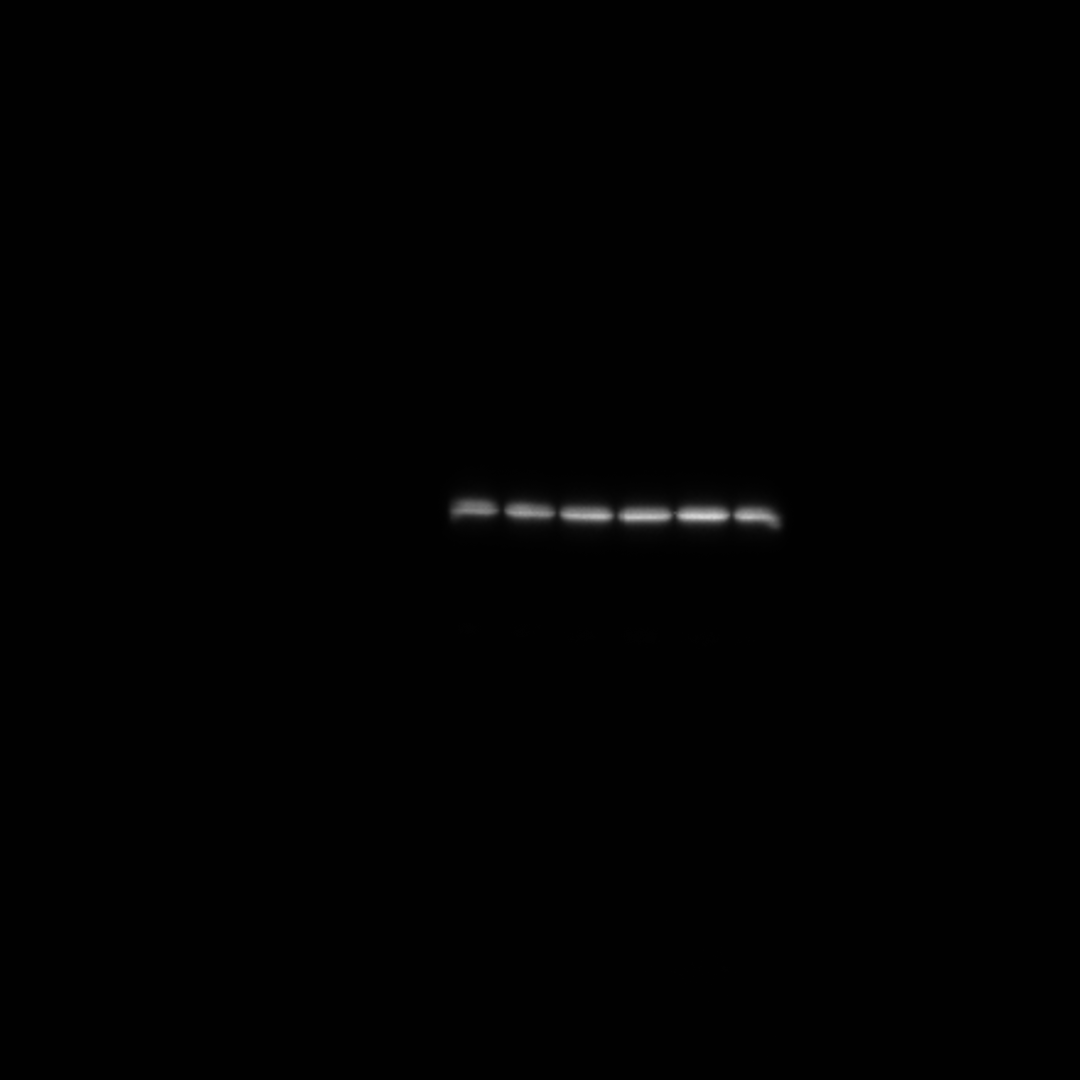

Supplement: Figure 2—source data 1. [file elife-99650-fig2-data1.zip › Figure 2 - Source Data 1/Figure2E_Tubulin_raw.Tif]

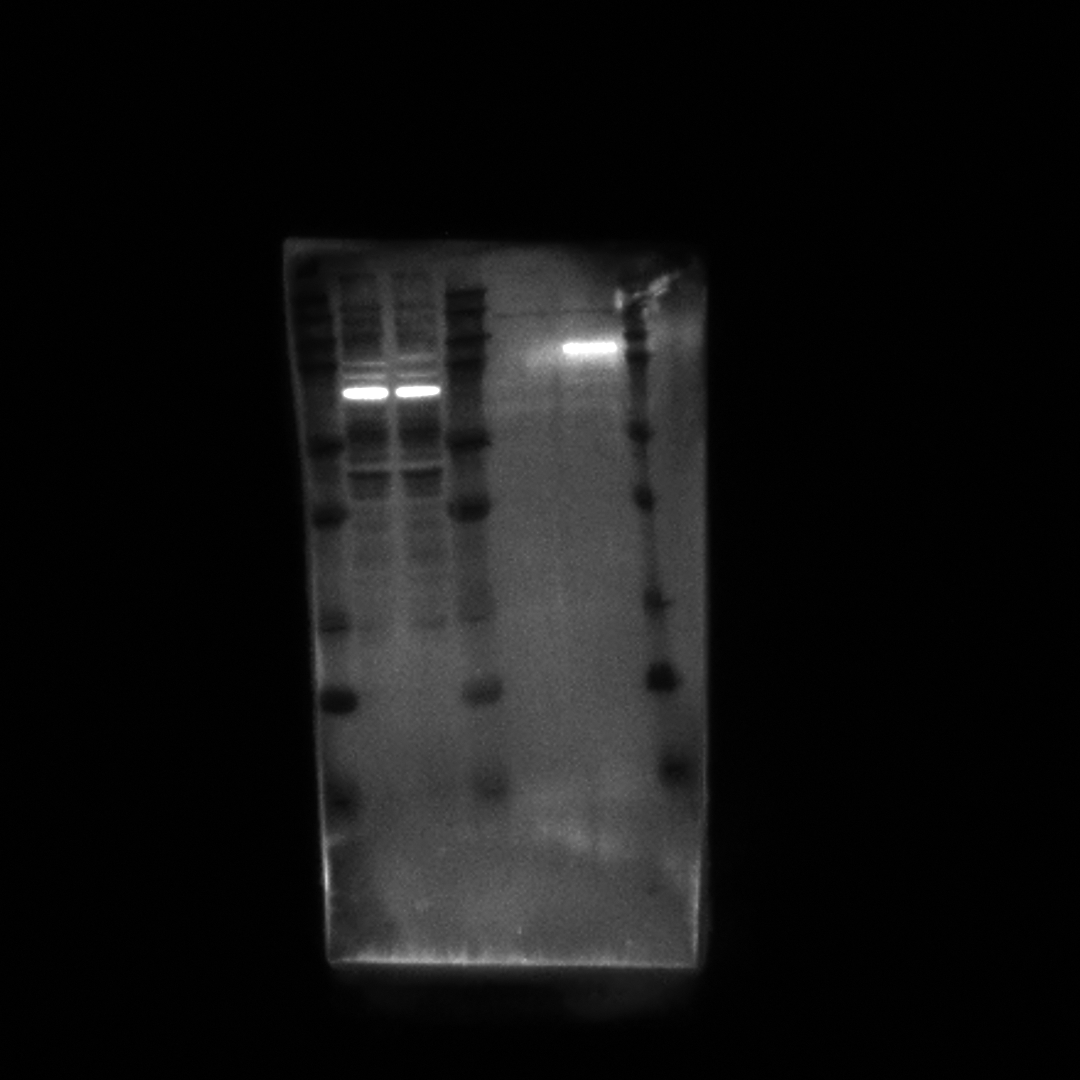

Supplement: Figure 2—source data 1. [file elife-99650-fig2-data1.zip › Figure 2 - Source Data 1/Figure2F_Fas3_raw.Tif]

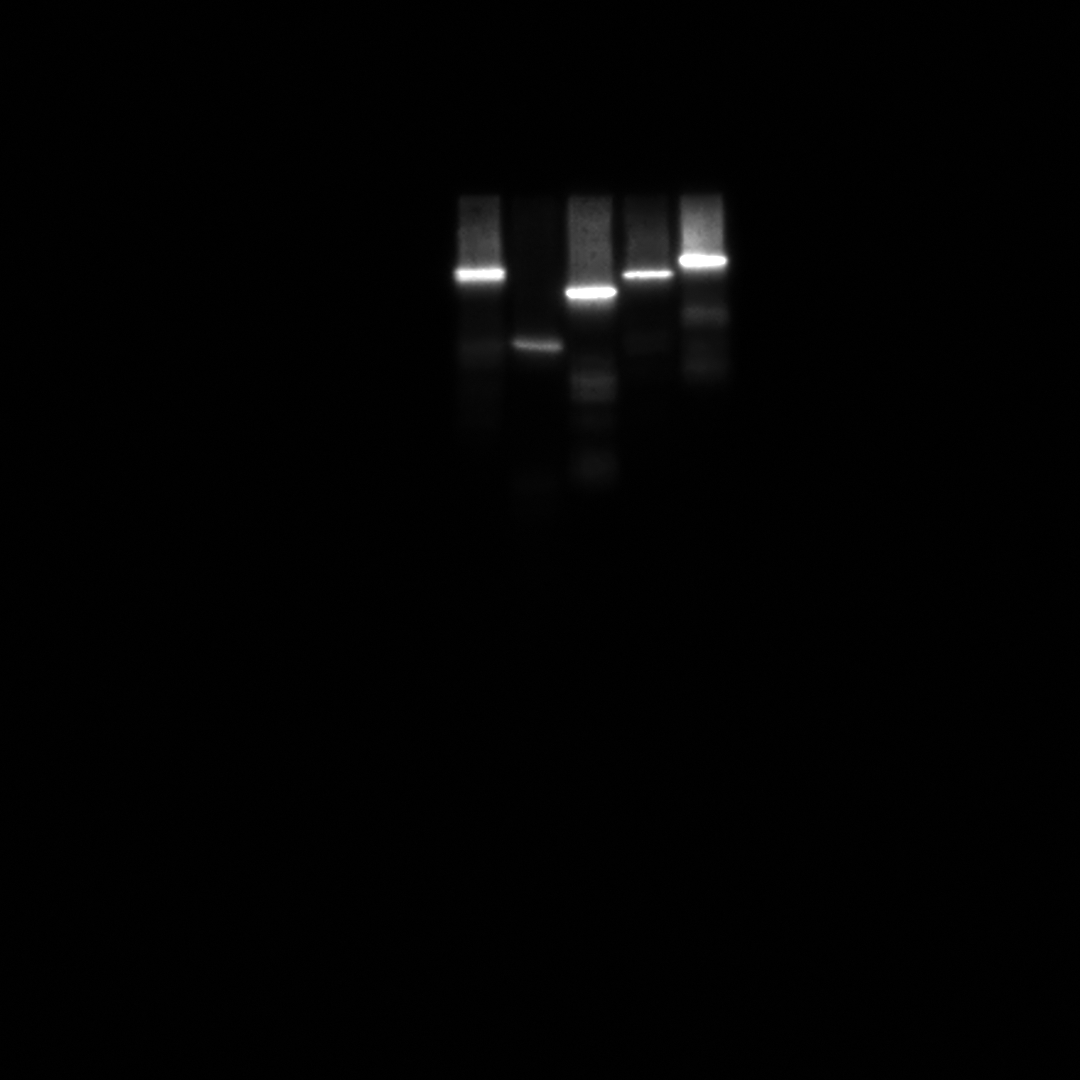

Supplement: Figure 2—source data 1. [file elife-99650-fig2-data1.zip › Figure 2 - Source Data 1/Figure2E_FLAG_raw.Tif]

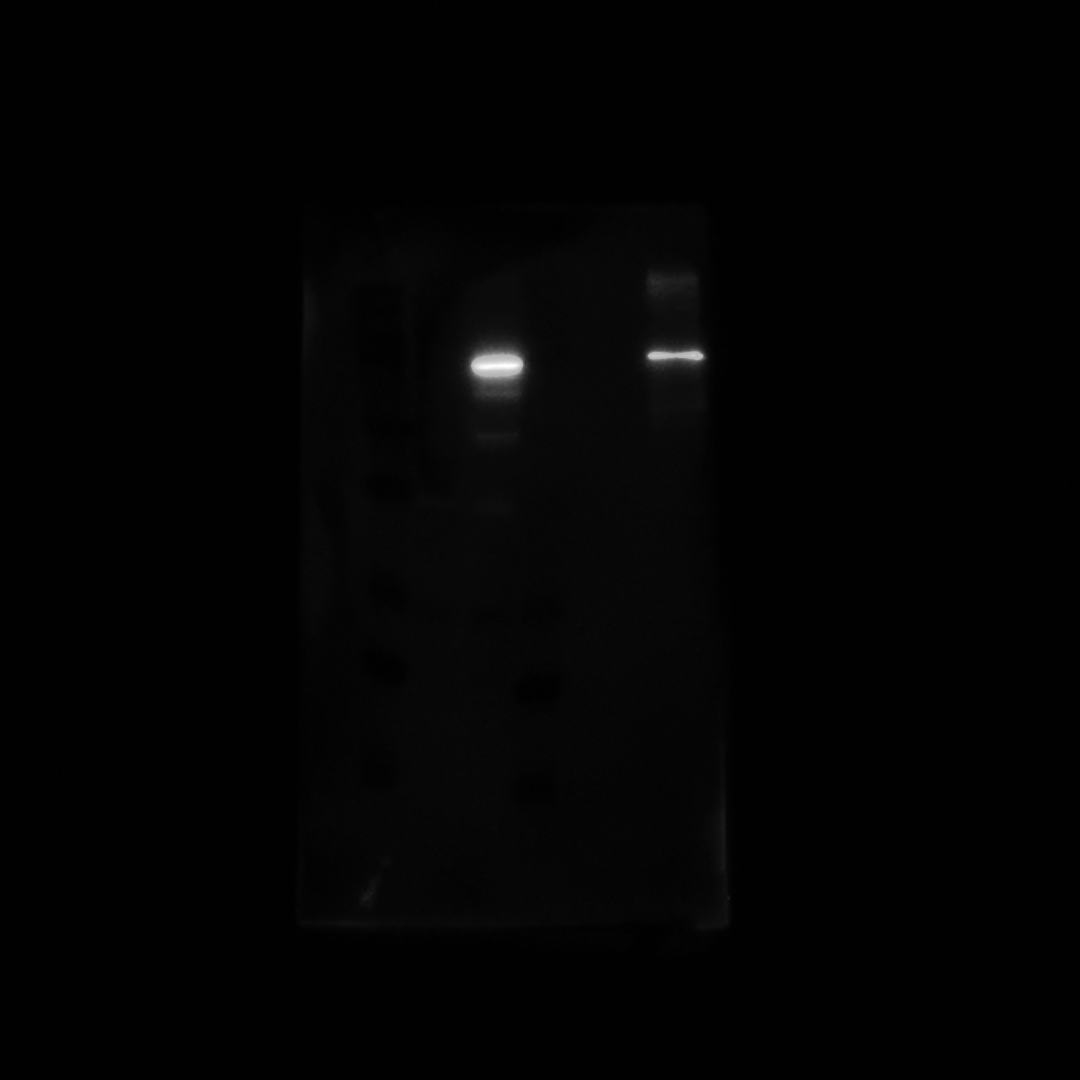

Supplement: Figure 2—source data 1. [file elife-99650-fig2-data1.zip › Figure 2 - Source Data 1/Figure2F_V5_raw.Tif]

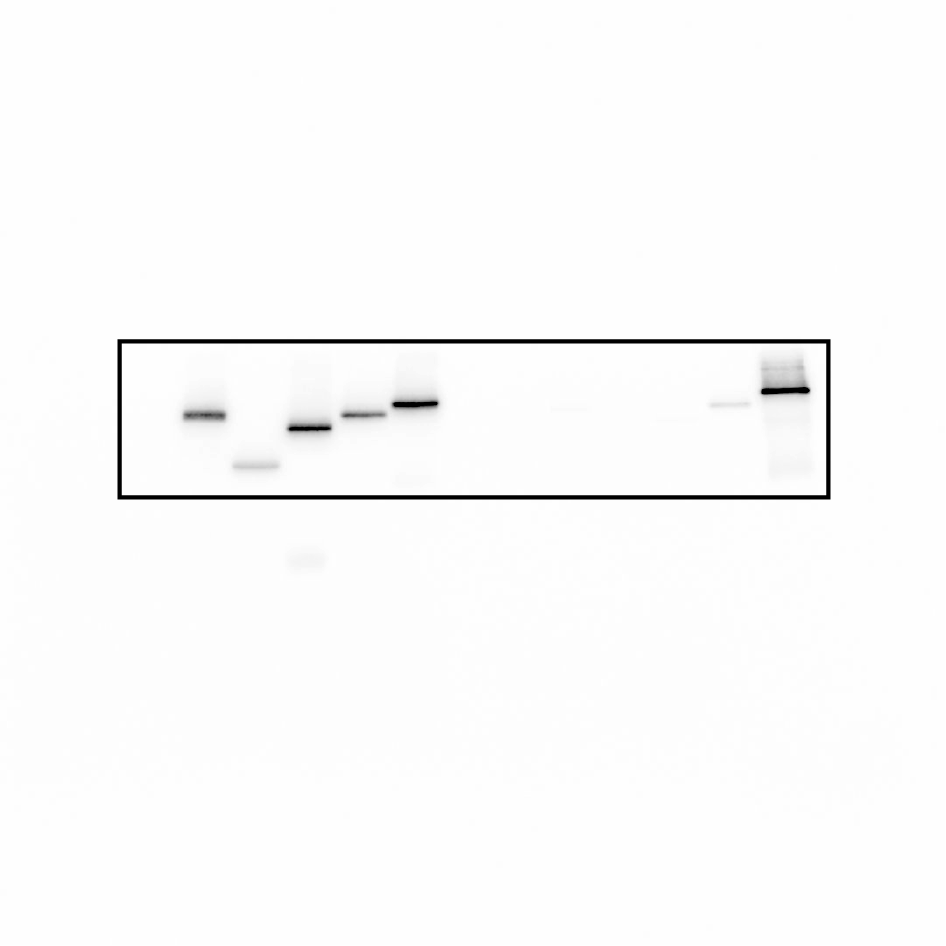

Supplement: Figure 2—source data 2. [file elife-99650-fig2-data2.zip › Figure 2 - Source Data 2/Figure2G_FLAG_annotated.tif]

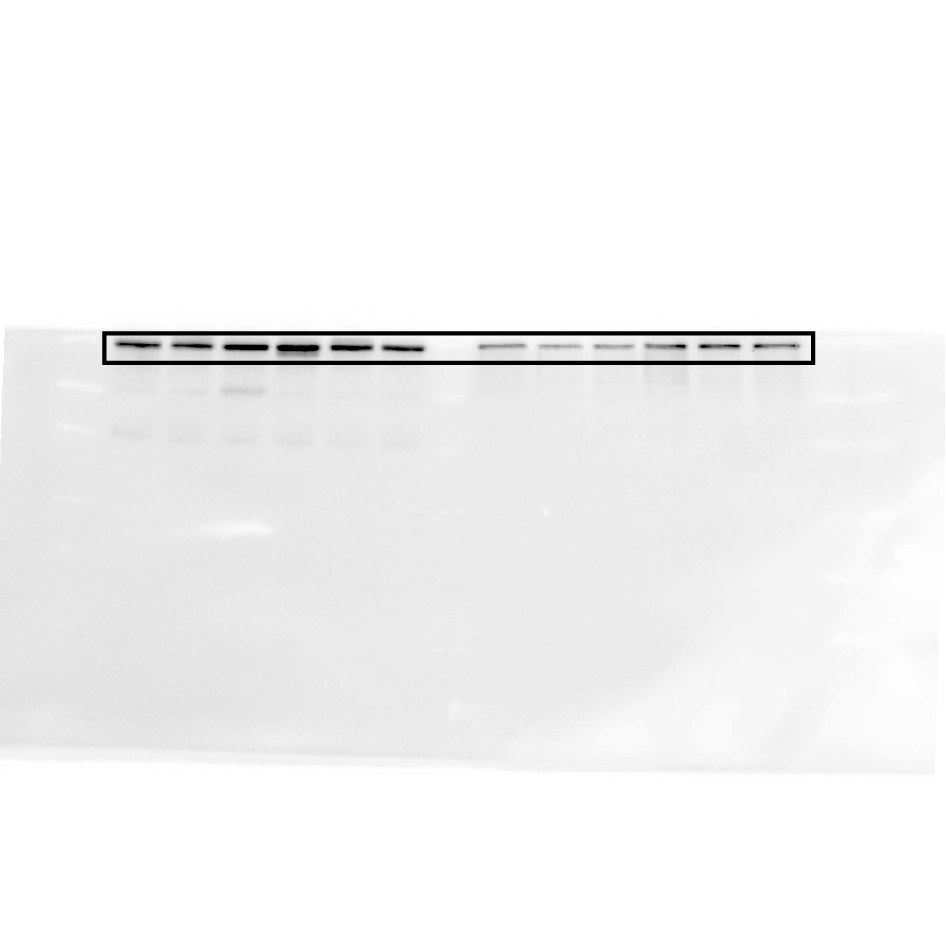

Supplement: Figure 2—source data 2. [file elife-99650-fig2-data2.zip › Figure 2 - Source Data 2/Figure2G_V5_annotated.tif]

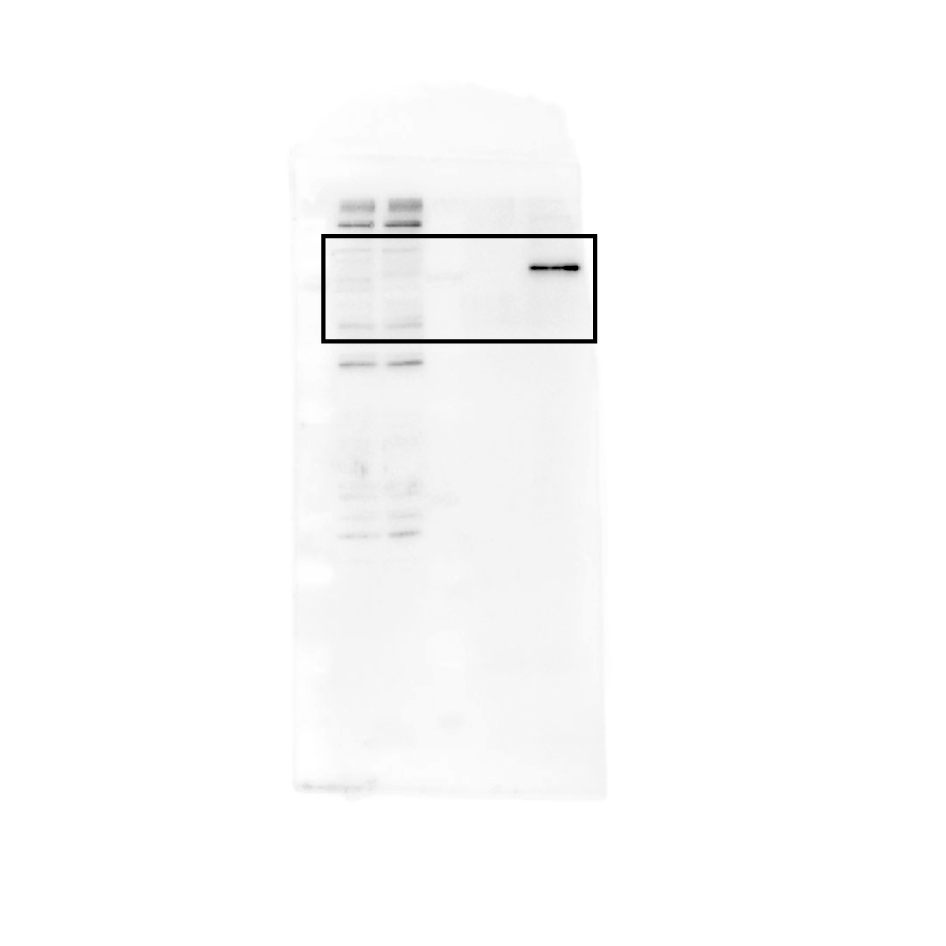

Supplement: Figure 2—source data 2. [file elife-99650-fig2-data2.zip › Figure 2 - Source Data 2/Figure2F_Fas3G_annotated.tif]

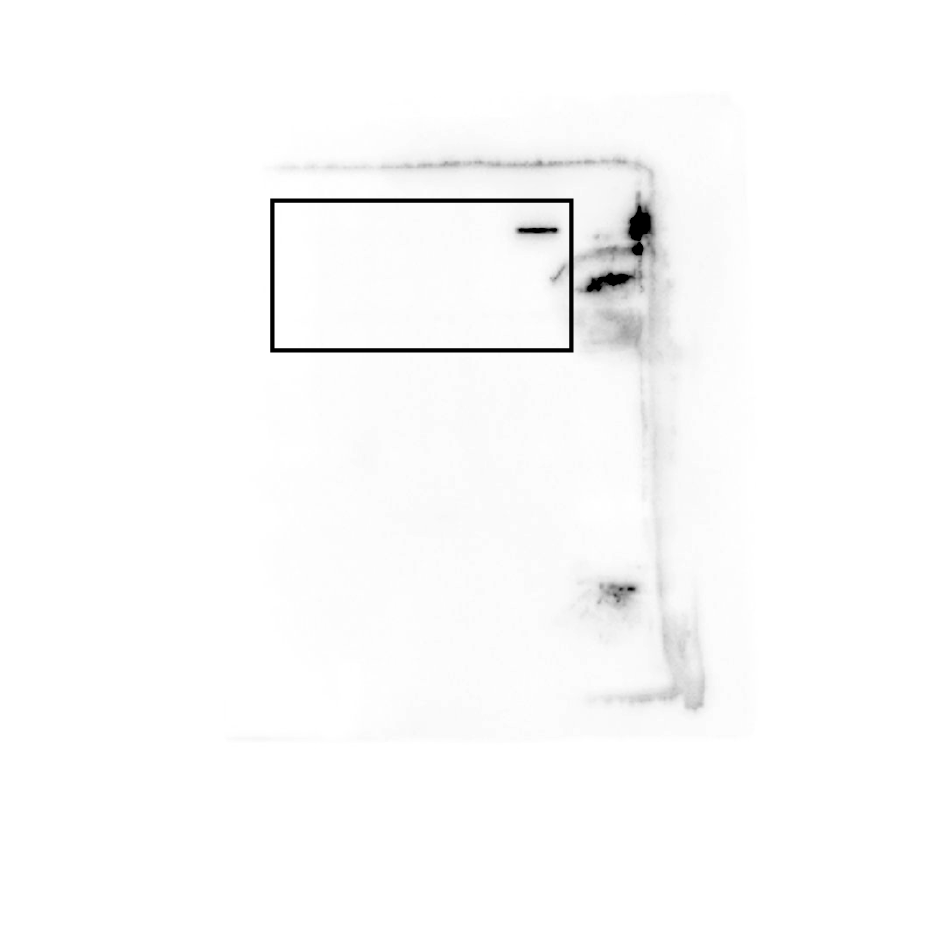

Supplement: Figure 2—source data 2. [file elife-99650-fig2-data2.zip › Figure 2 - Source Data 2/Figure2E_Fas3G_annotated.tif]

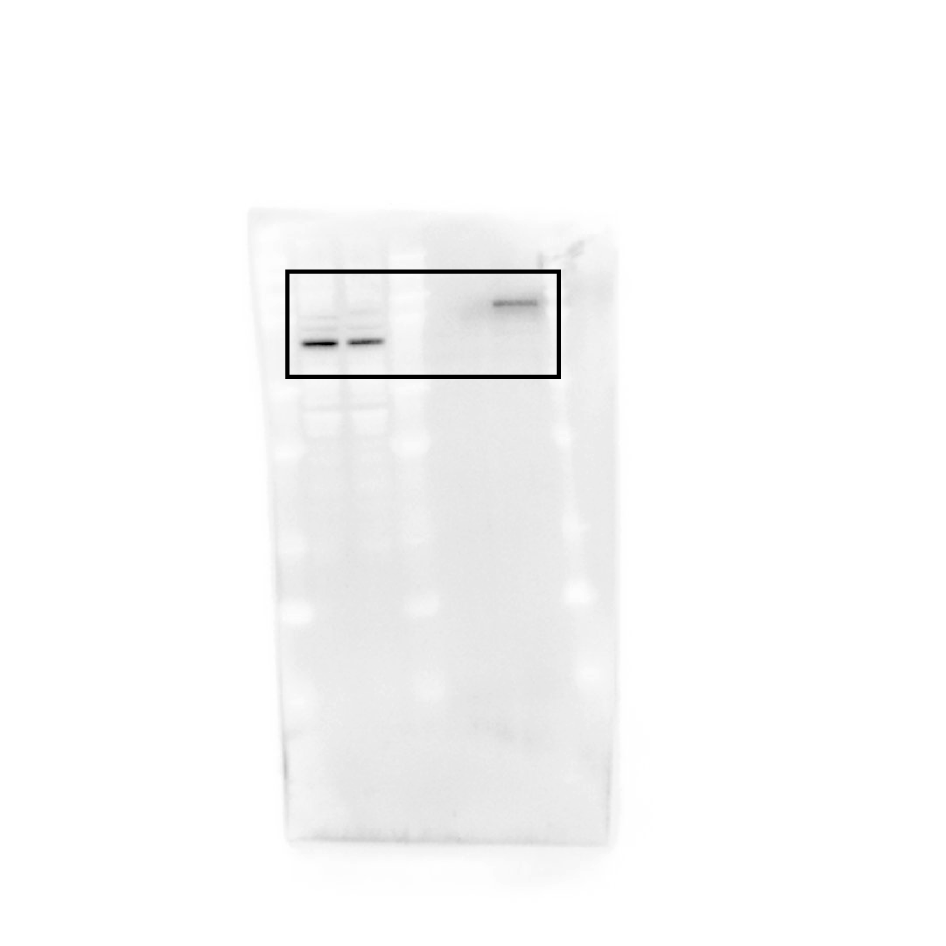

Supplement: Figure 2—source data 2. [file elife-99650-fig2-data2.zip › Figure 2 - Source Data 2/Figure2F_Fas3_annotated.tif]

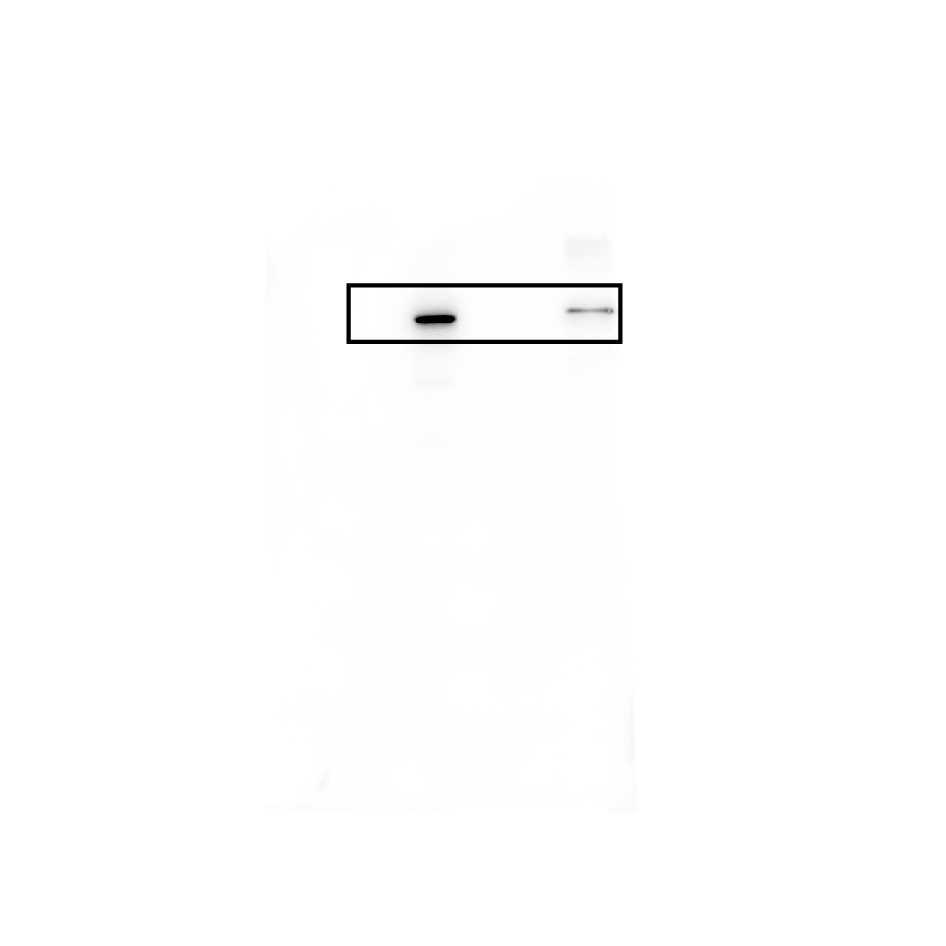

Supplement: Figure 2—source data 2. [file elife-99650-fig2-data2.zip › Figure 2 - Source Data 2/Figure2F_V5_annotated.tif]

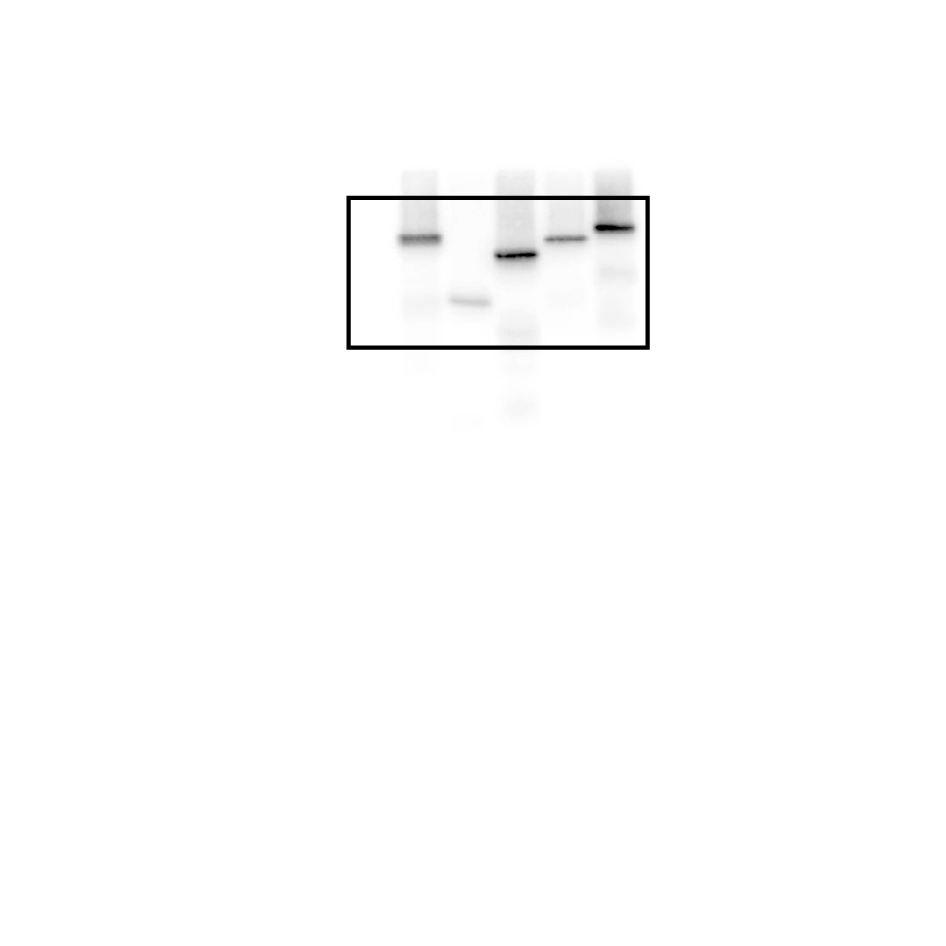

Supplement: Figure 2—source data 2. [file elife-99650-fig2-data2.zip › Figure 2 - Source Data 2/Figure2E_FLAG_annotated.tif]

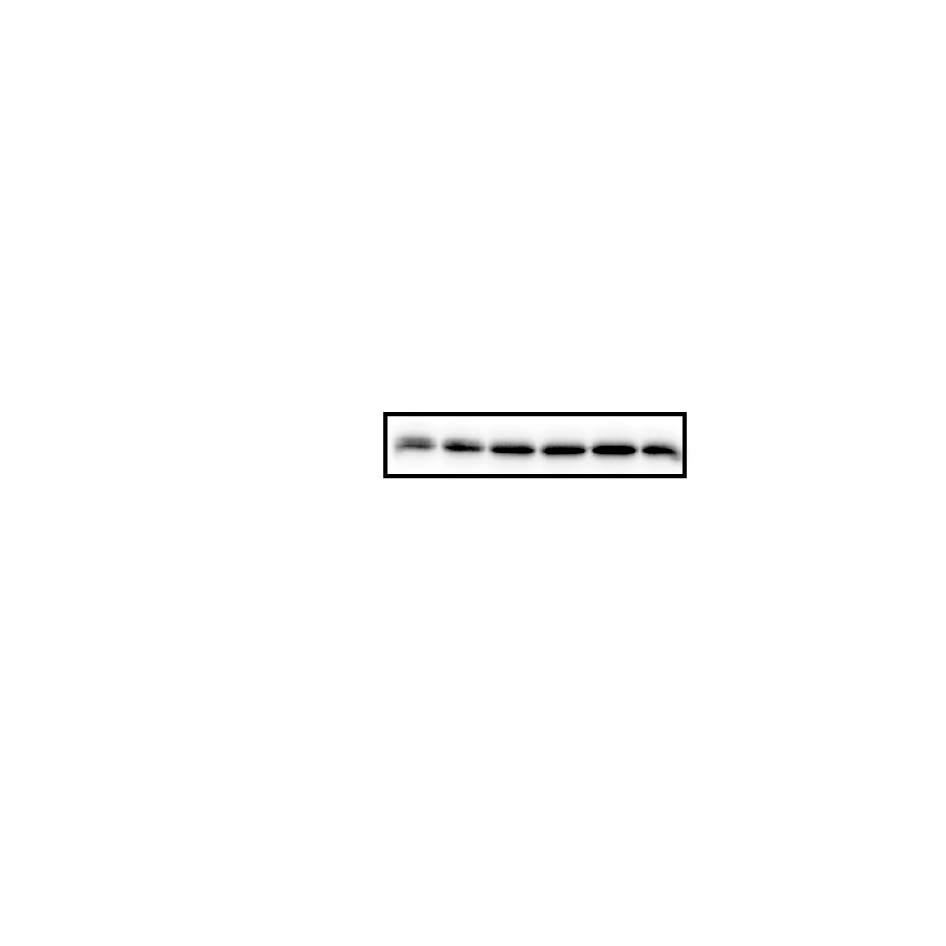

Supplement: Figure 2—source data 2. [file elife-99650-fig2-data2.zip › Figure 2 - Source Data 2/Figure2E_Tubulin_annotated.tif]

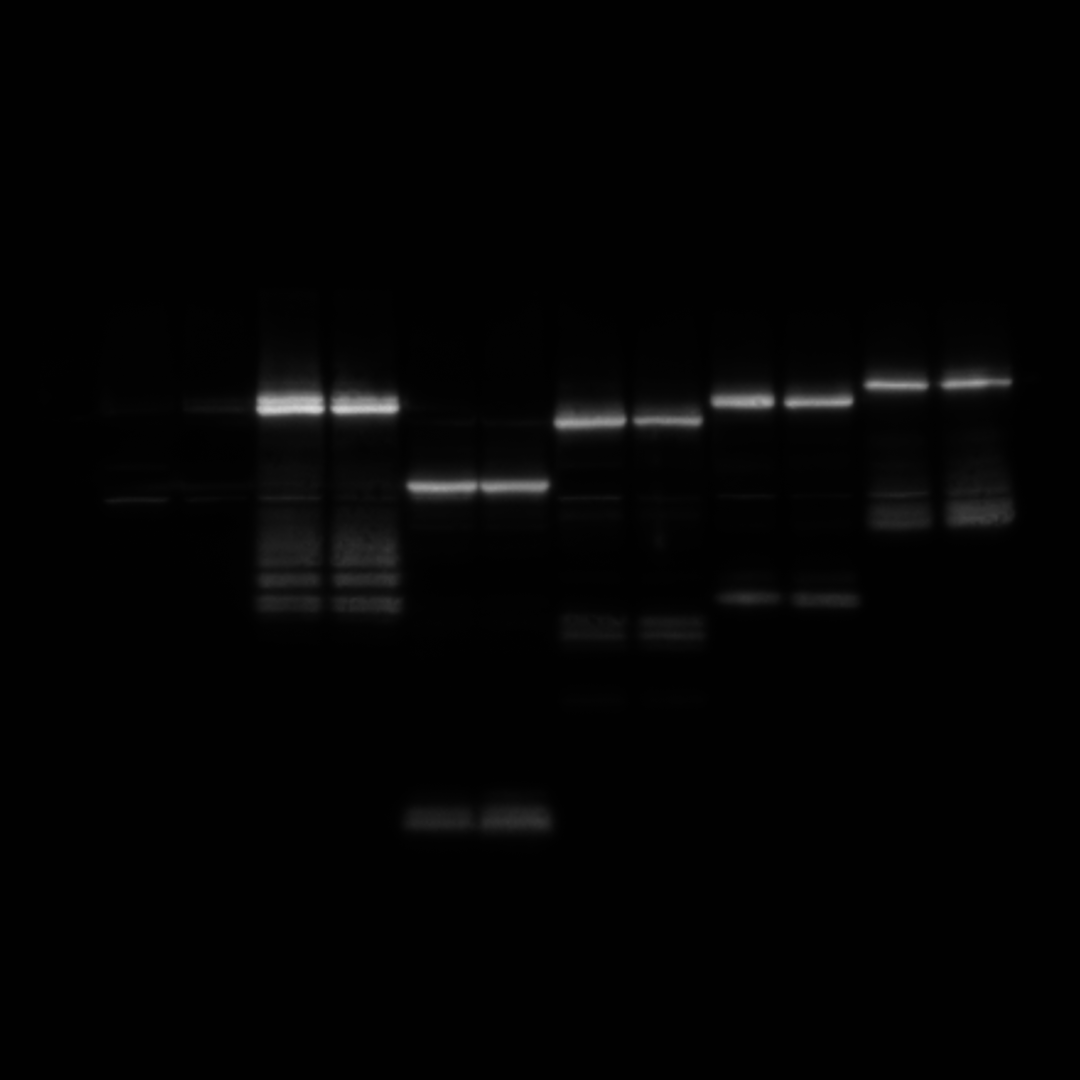

Supplement: Figure 2—figure supplement 2—source data 1. [file elife-99650-fig2-figsupp2-data1.zip › Figure 2 - figure supplement 2 - Source Data 1/Figure2_figuresupplement2B_FLAG_raw.Tif]

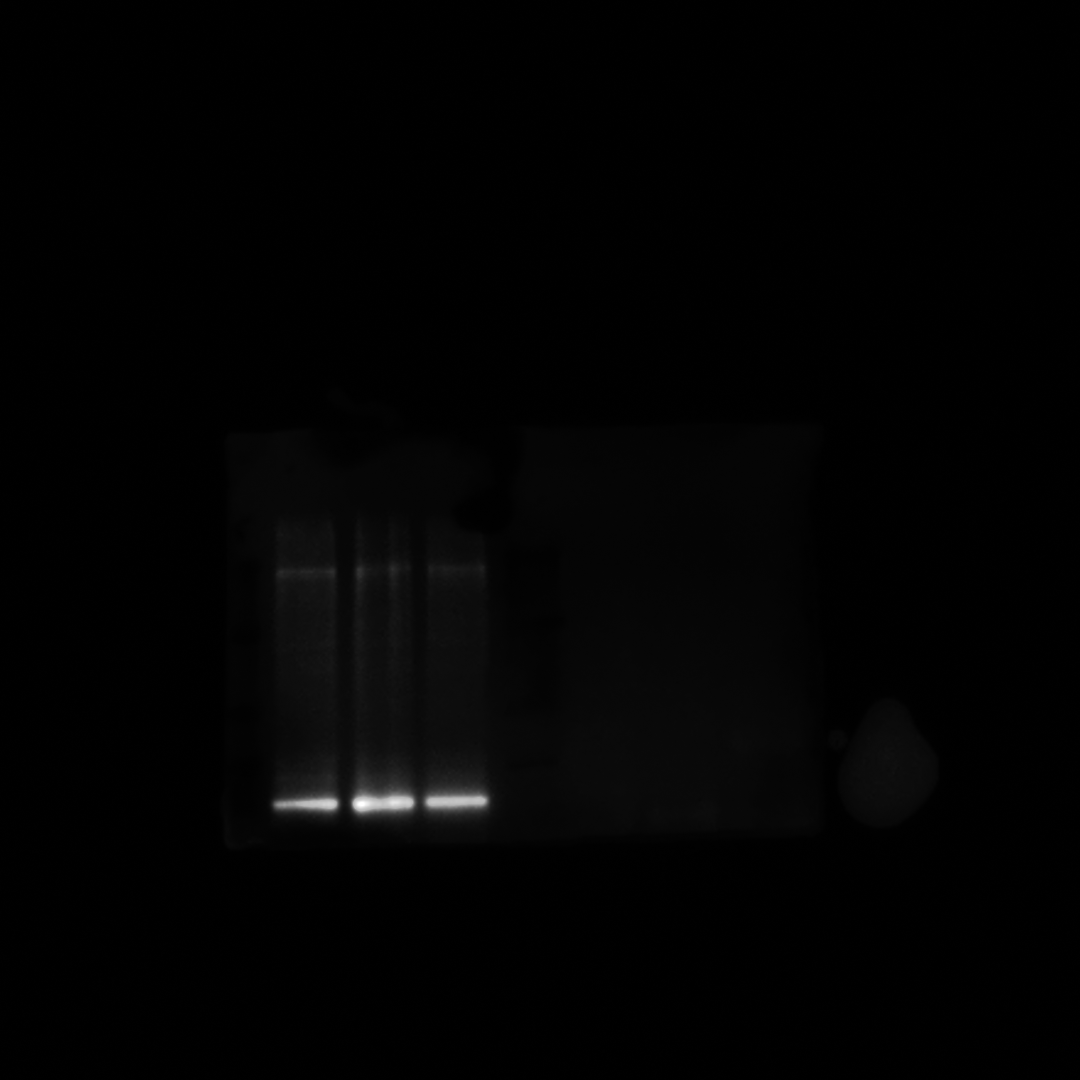

Supplement: Figure 2—figure supplement 2—source data 1. [file elife-99650-fig2-figsupp2-data1.zip › Figure 2 - figure supplement 2 - Source Data 1/Figure2_figuresupplement2A_V5_raw.Tif]

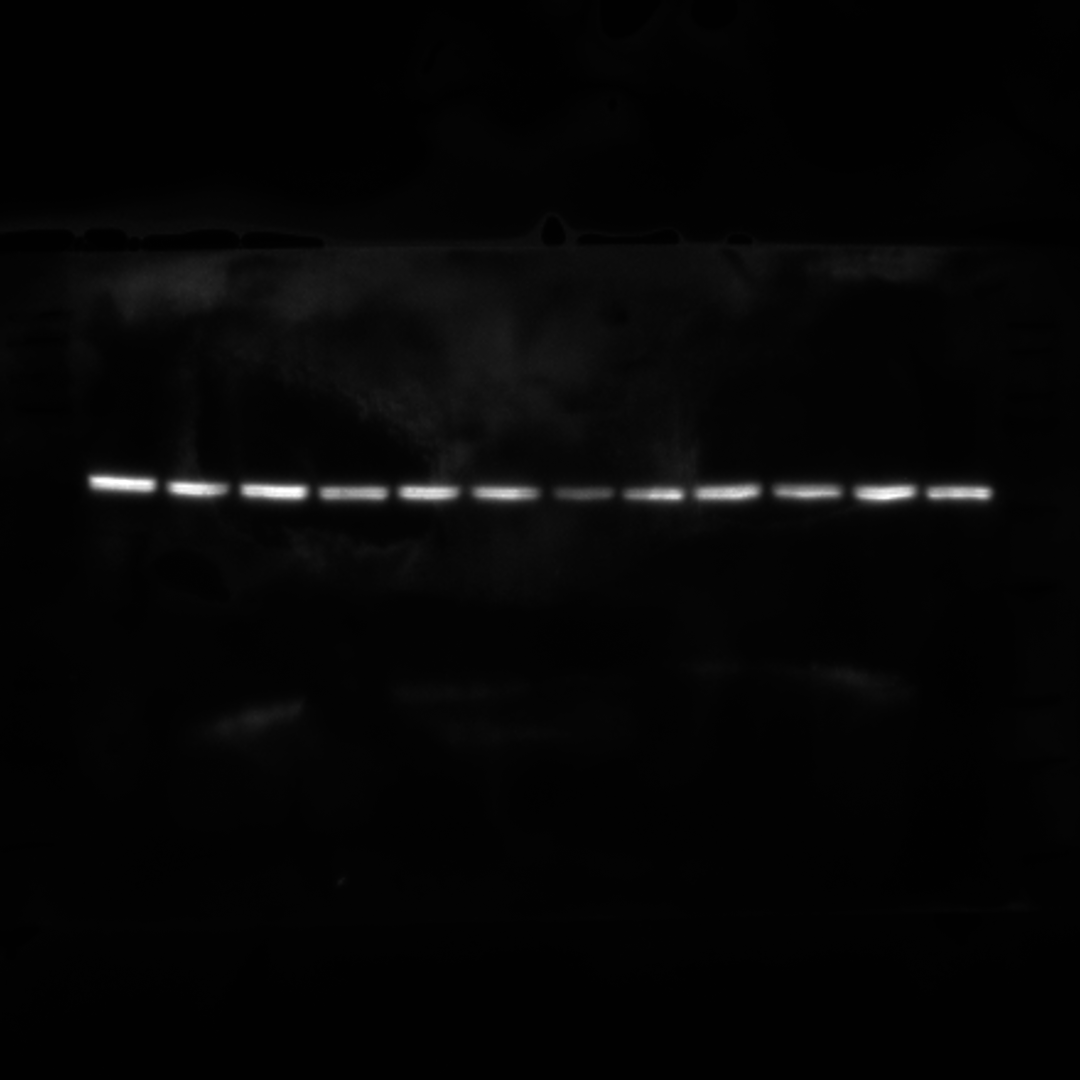

Supplement: Figure 2—figure supplement 2—source data 1. [file elife-99650-fig2-figsupp2-data1.zip › Figure 2 - figure supplement 2 - Source Data 1/Figure2_figuresupplement2B_Tubulin_raw.Tif]

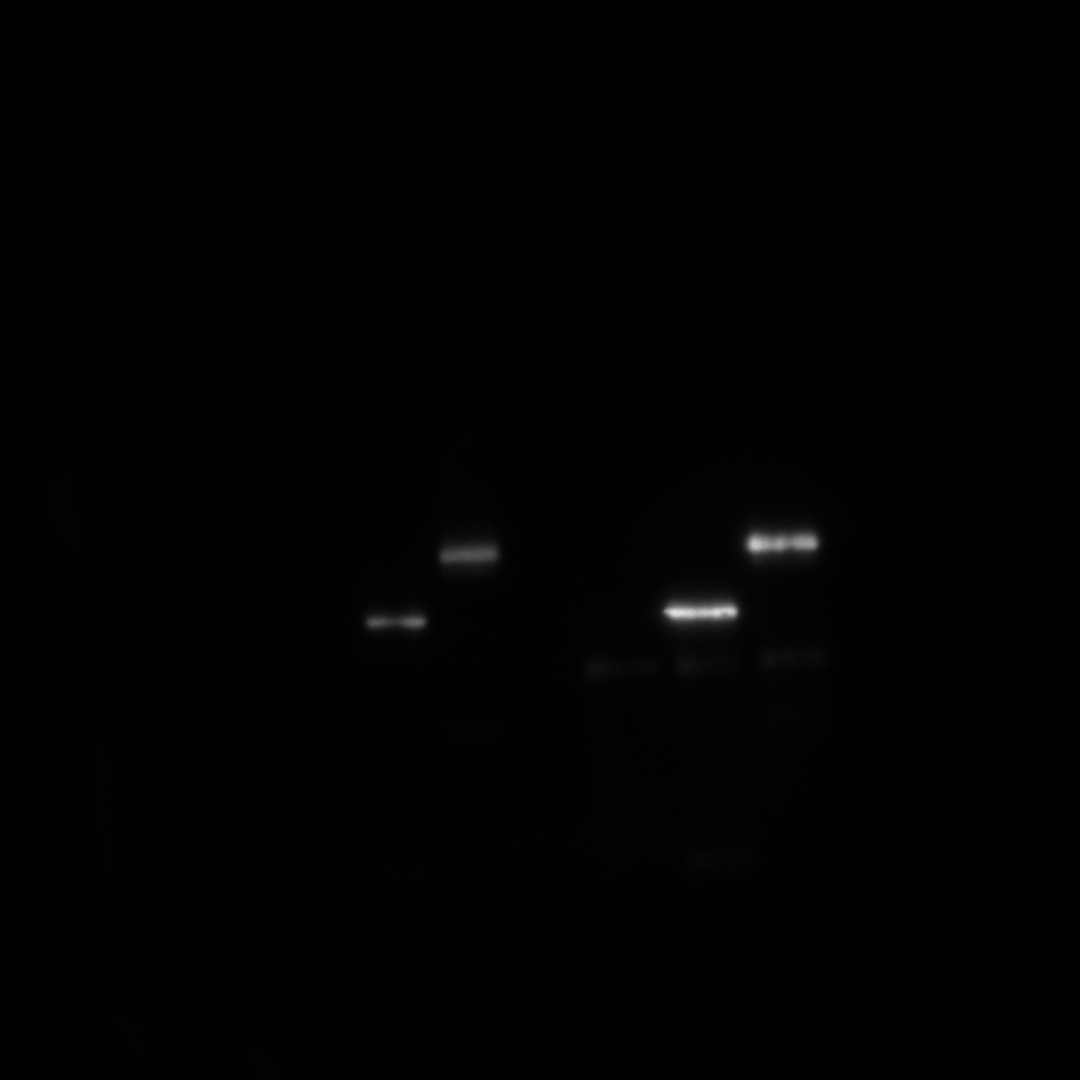

Supplement: Figure 2—figure supplement 2—source data 1. [file elife-99650-fig2-figsupp2-data1.zip › Figure 2 - figure supplement 2 - Source Data 1/Figure2_figuresupplement2A_FLAG_raw.Tif]

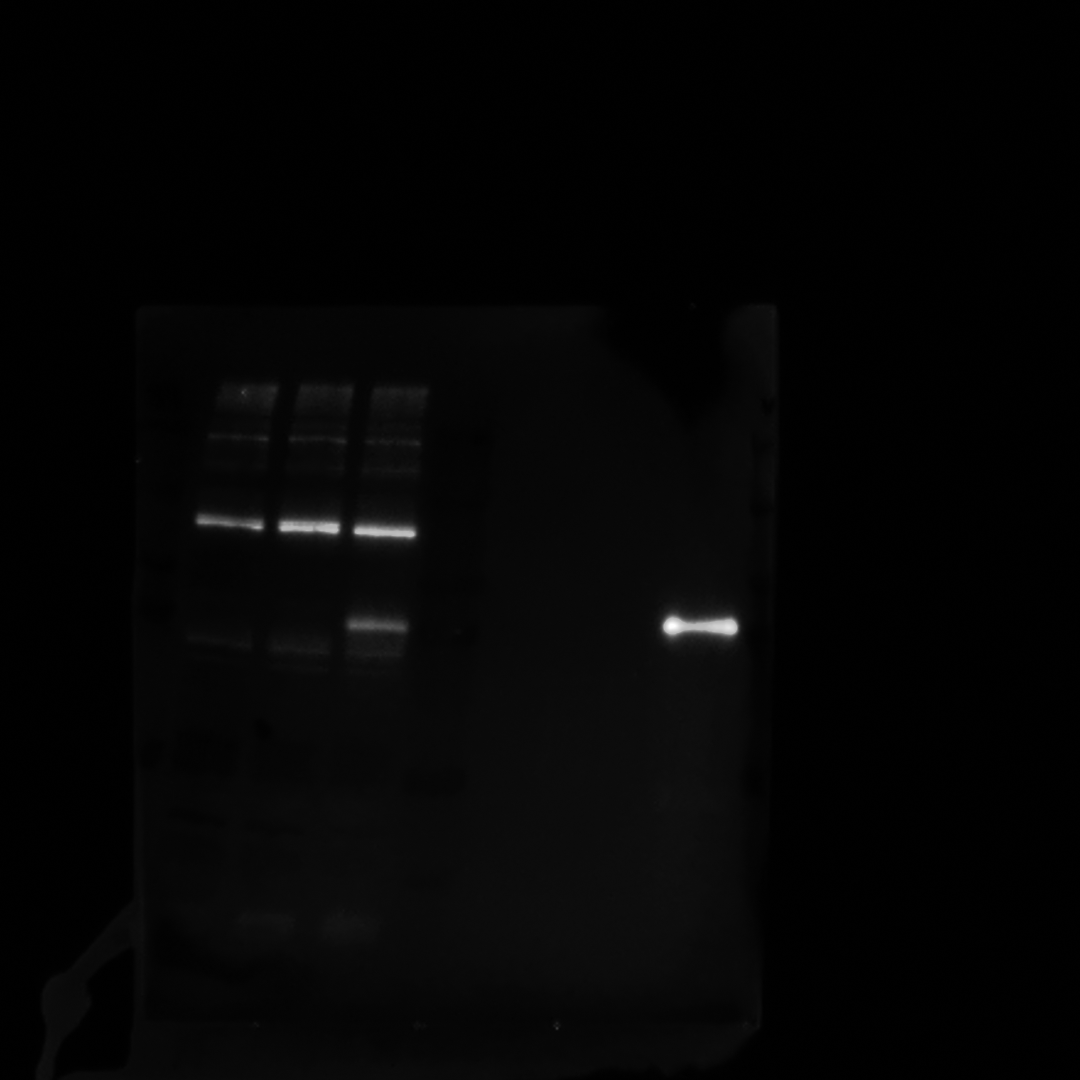

Supplement: Figure 2—figure supplement 2—source data 1. [file elife-99650-fig2-figsupp2-data1.zip › Figure 2 - figure supplement 2 - Source Data 1/Figure2_figuresupplement2A_Streptavidin_raw.Tif]

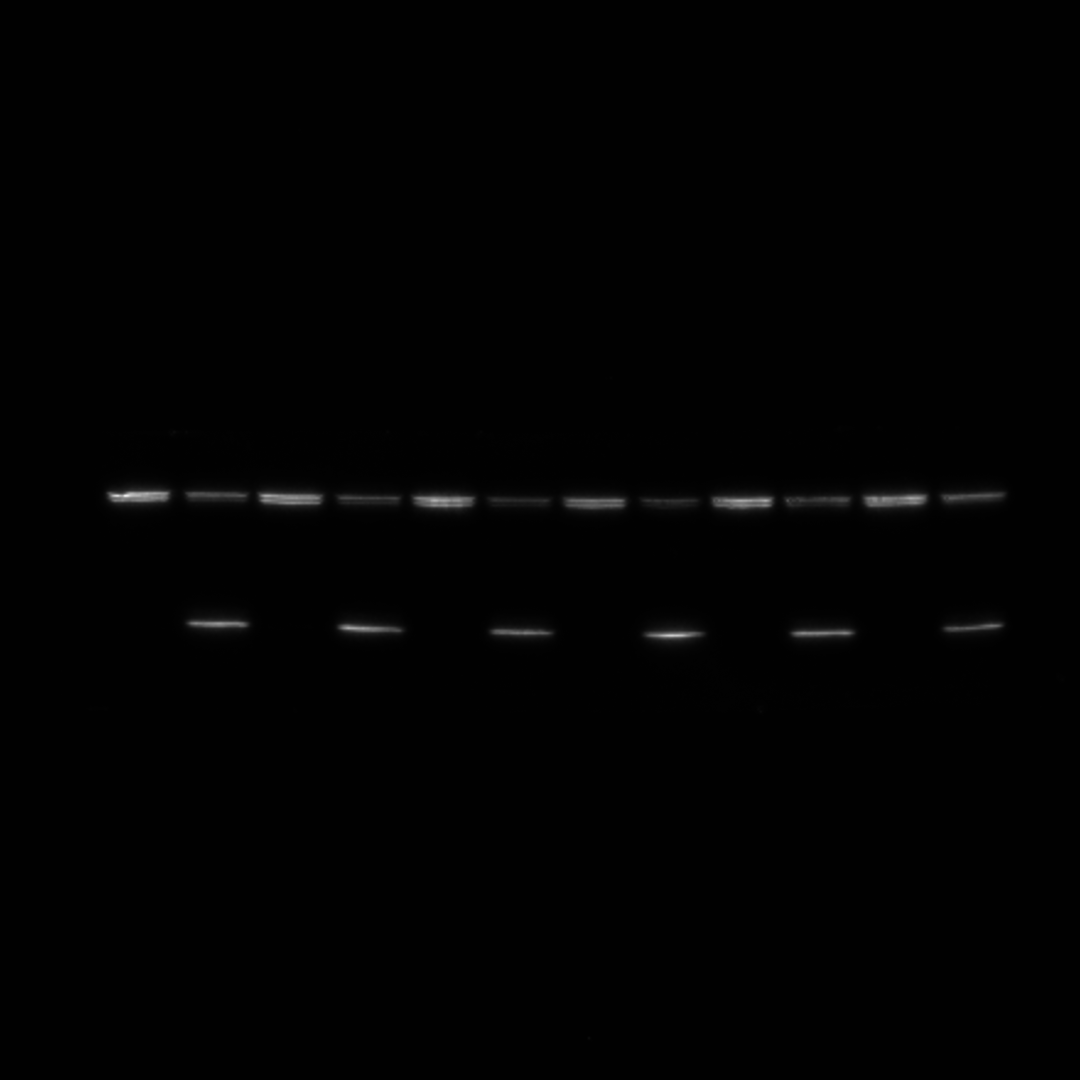

Supplement: Figure 2—figure supplement 2—source data 1. [file elife-99650-fig2-figsupp2-data1.zip › Figure 2 - figure supplement 2 - Source Data 1/Figure2_figuresupplement2B_LaminB_raw.Tif]

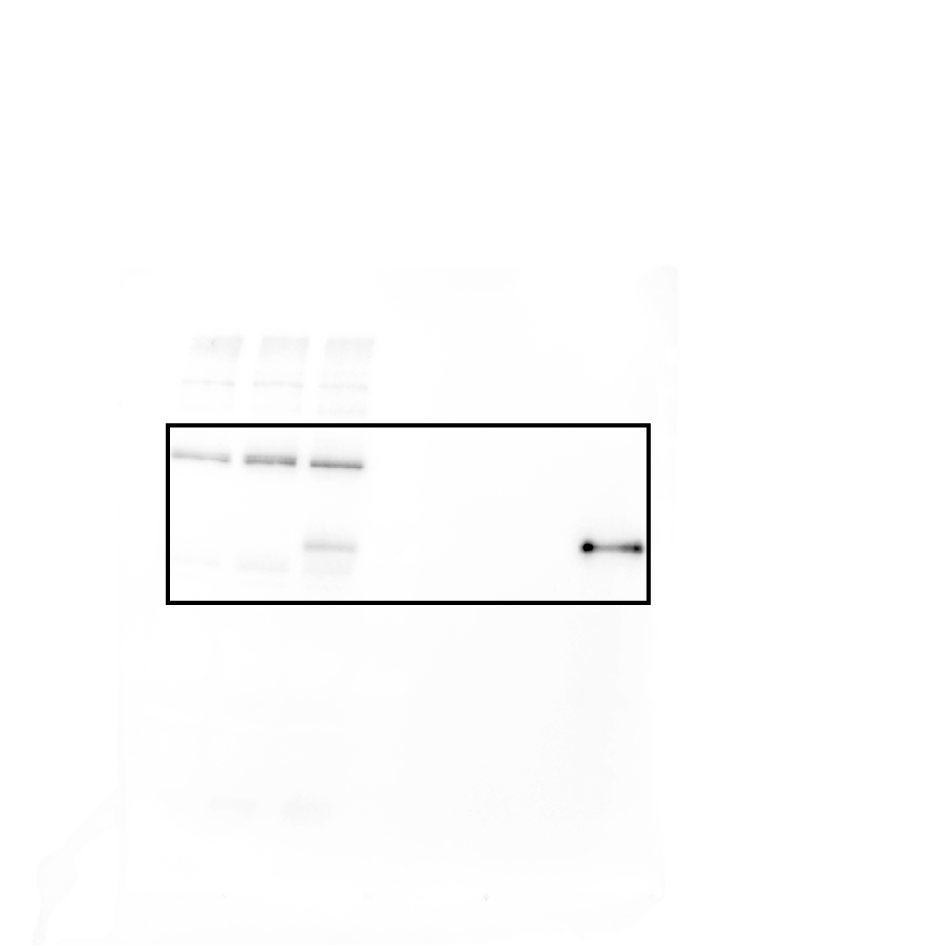

Supplement: Figure 2—figure supplement 2—source data 2. [file elife-99650-fig2-figsupp2-data2.zip › Figure 2 - figure supplement 2 - Source Data 2/Figure2_figuresupplement2A_Streptavidin_annotated.tif]

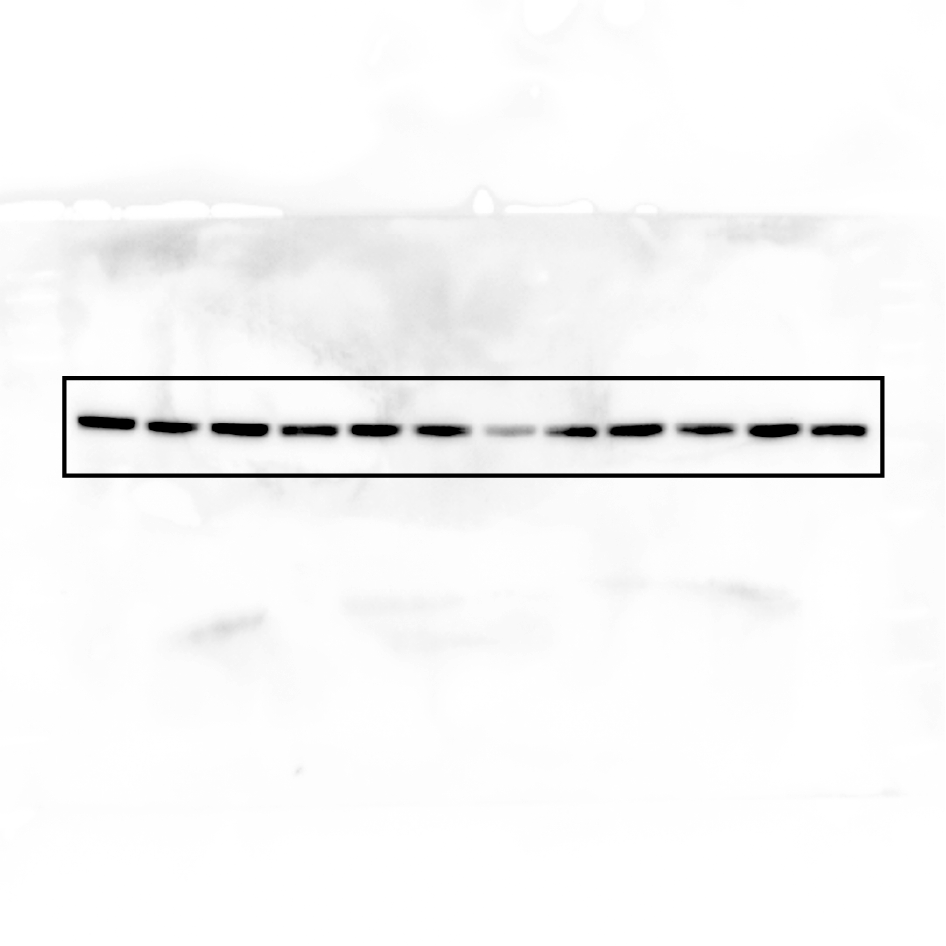

Supplement: Figure 2—figure supplement 2—source data 2. [file elife-99650-fig2-figsupp2-data2.zip › Figure 2 - figure supplement 2 - Source Data 2/Figure2_figuresupplement2B_Tubulin_annotated.tif]

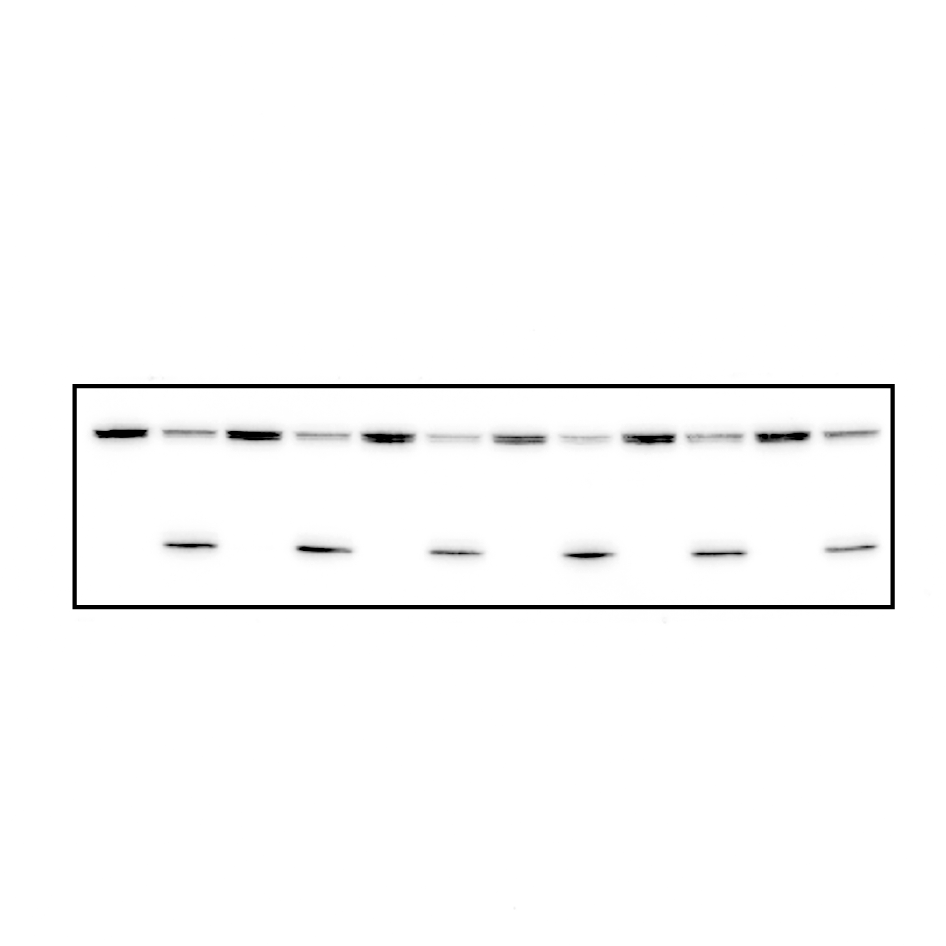

Supplement: Figure 2—figure supplement 2—source data 2. [file elife-99650-fig2-figsupp2-data2.zip › Figure 2 - figure supplement 2 - Source Data 2/Figure2_figuresupplement2B_LaminB_annotated.tif]

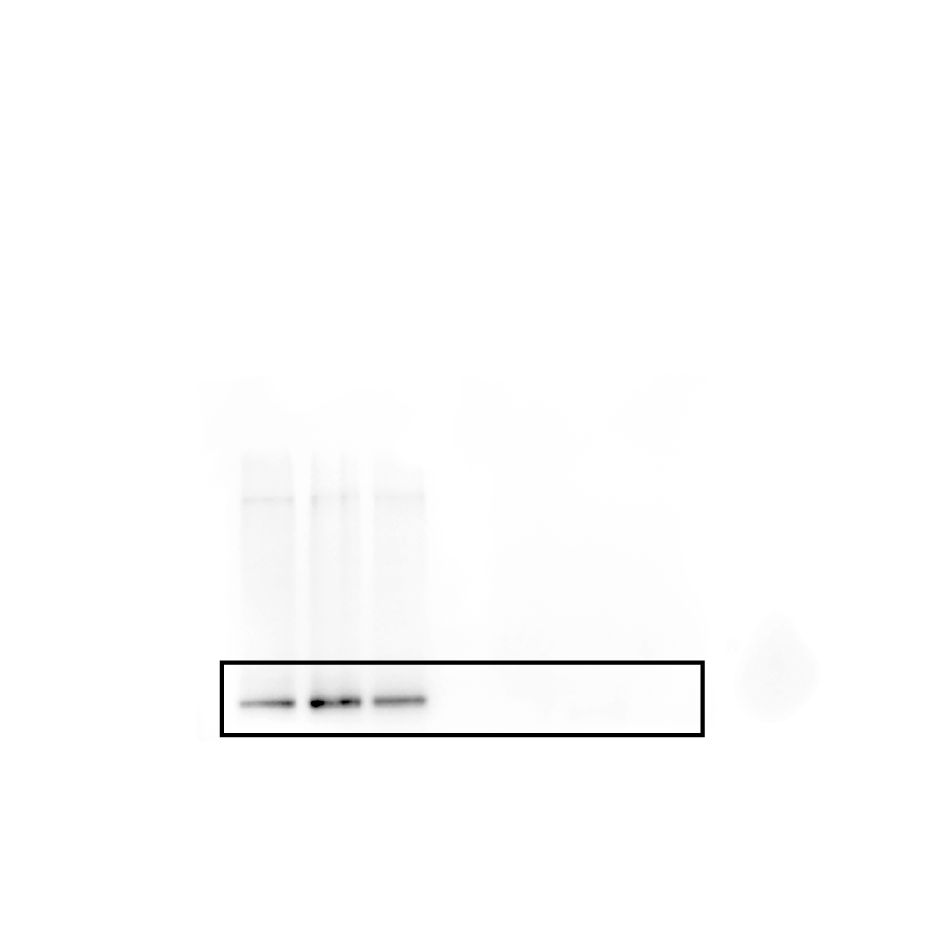

Supplement: Figure 2—figure supplement 2—source data 2. [file elife-99650-fig2-figsupp2-data2.zip › Figure 2 - figure supplement 2 - Source Data 2/Figure2_figuresupplement2A_V5_annotated.tif]

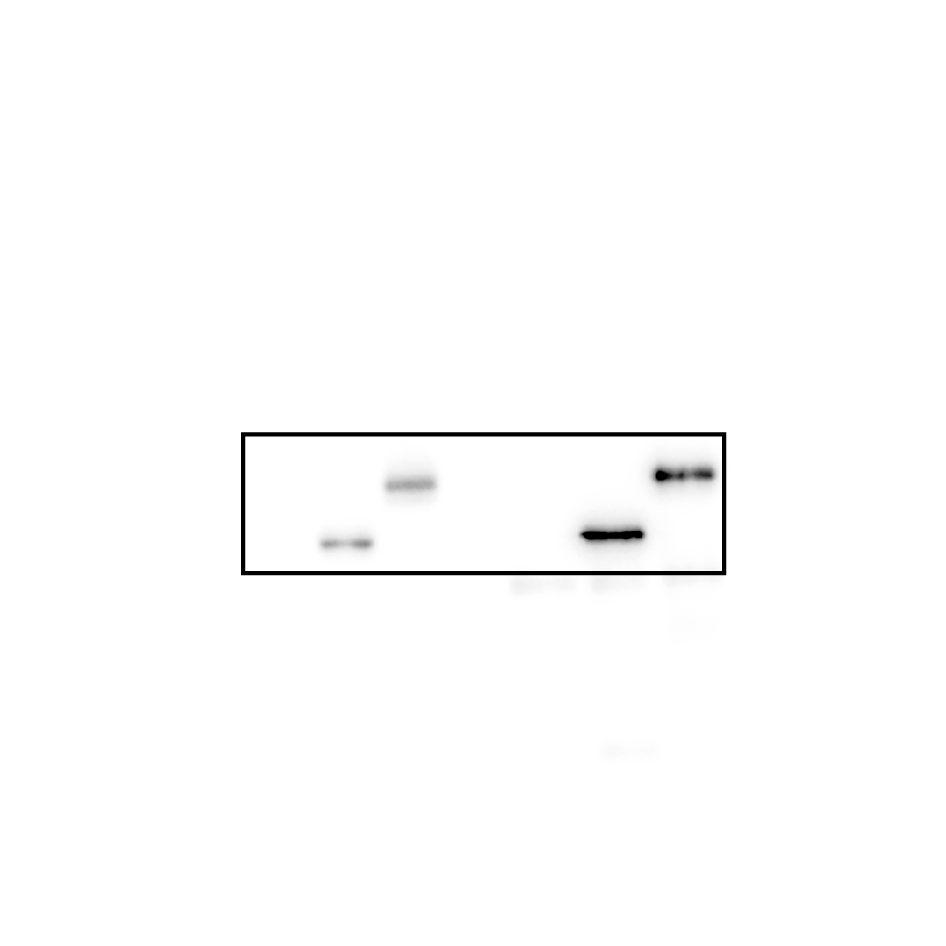

Supplement: Figure 2—figure supplement 2—source data 2. [file elife-99650-fig2-figsupp2-data2.zip › Figure 2 - figure supplement 2 - Source Data 2/Figure2_figuresupplement2A_FLAG_annotated.tif]

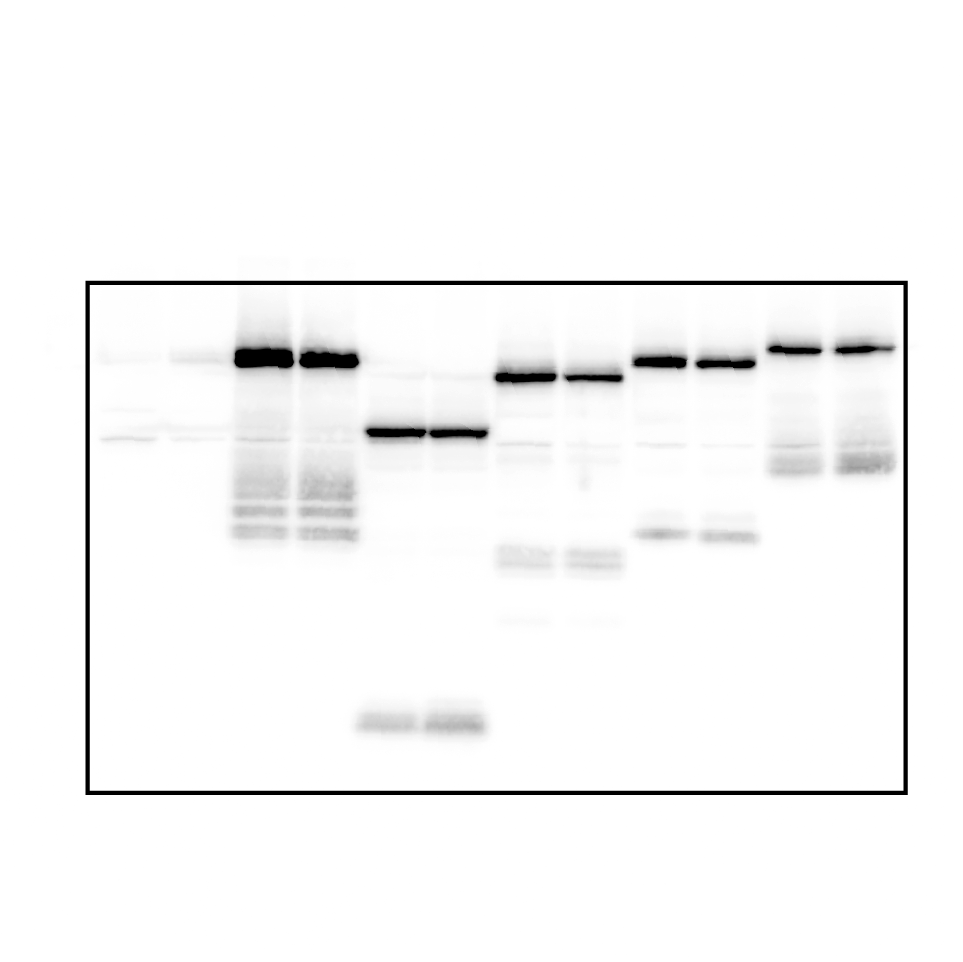

Supplement: Figure 2—figure supplement 2—source data 2. [file elife-99650-fig2-figsupp2-data2.zip › Figure 2 - figure supplement 2 - Source Data 2/Figure2_figuresupplement2B_FLAG_annotated.tif]

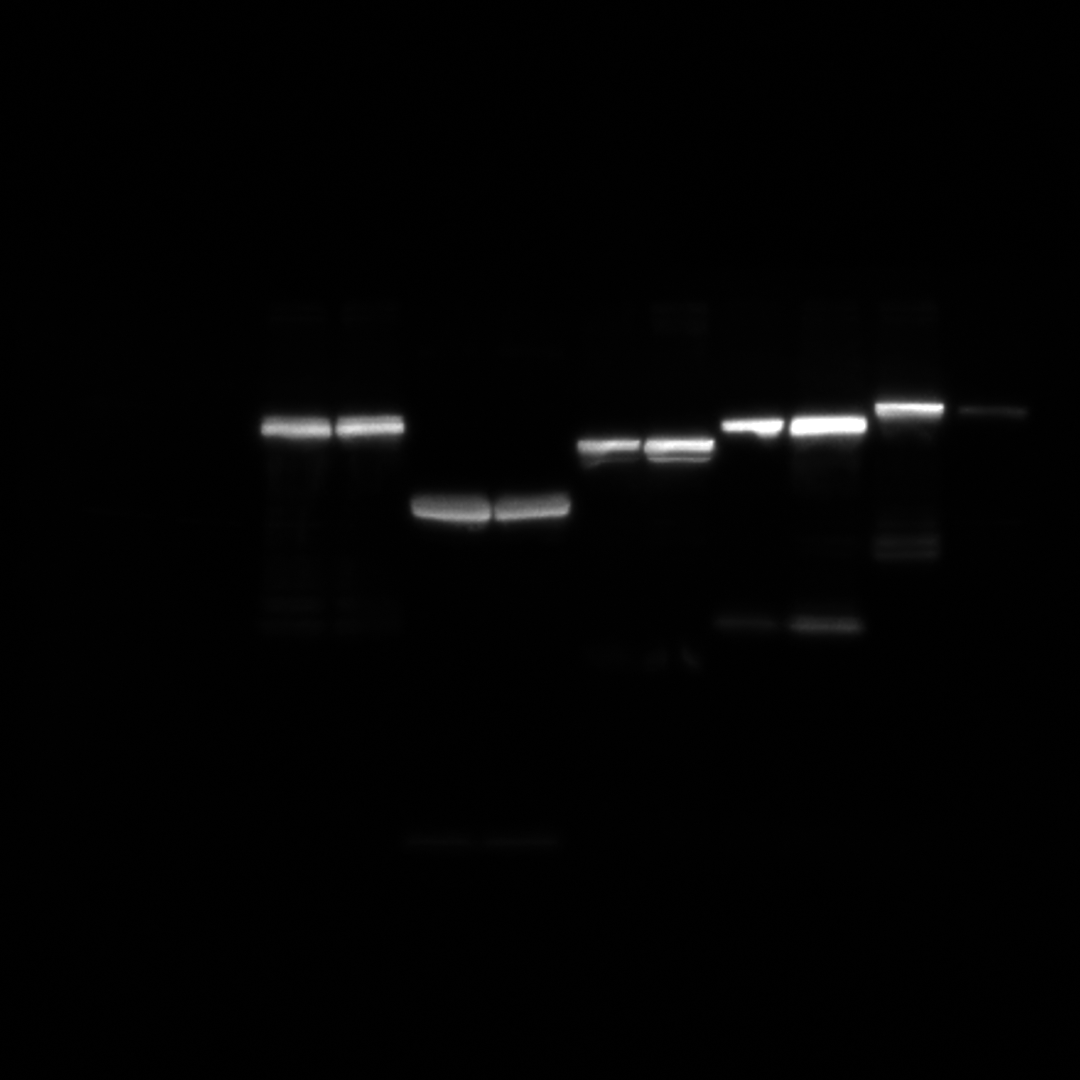

Supplement: Figure 4—figure supplement 1—source data 1. [file elife-99650-fig4-figsupp1-data1.zip › Figure 4 - figure supplement 1 - Source Data 1/Figure4_figuresupplement1B_FLAG_raw.Tif]

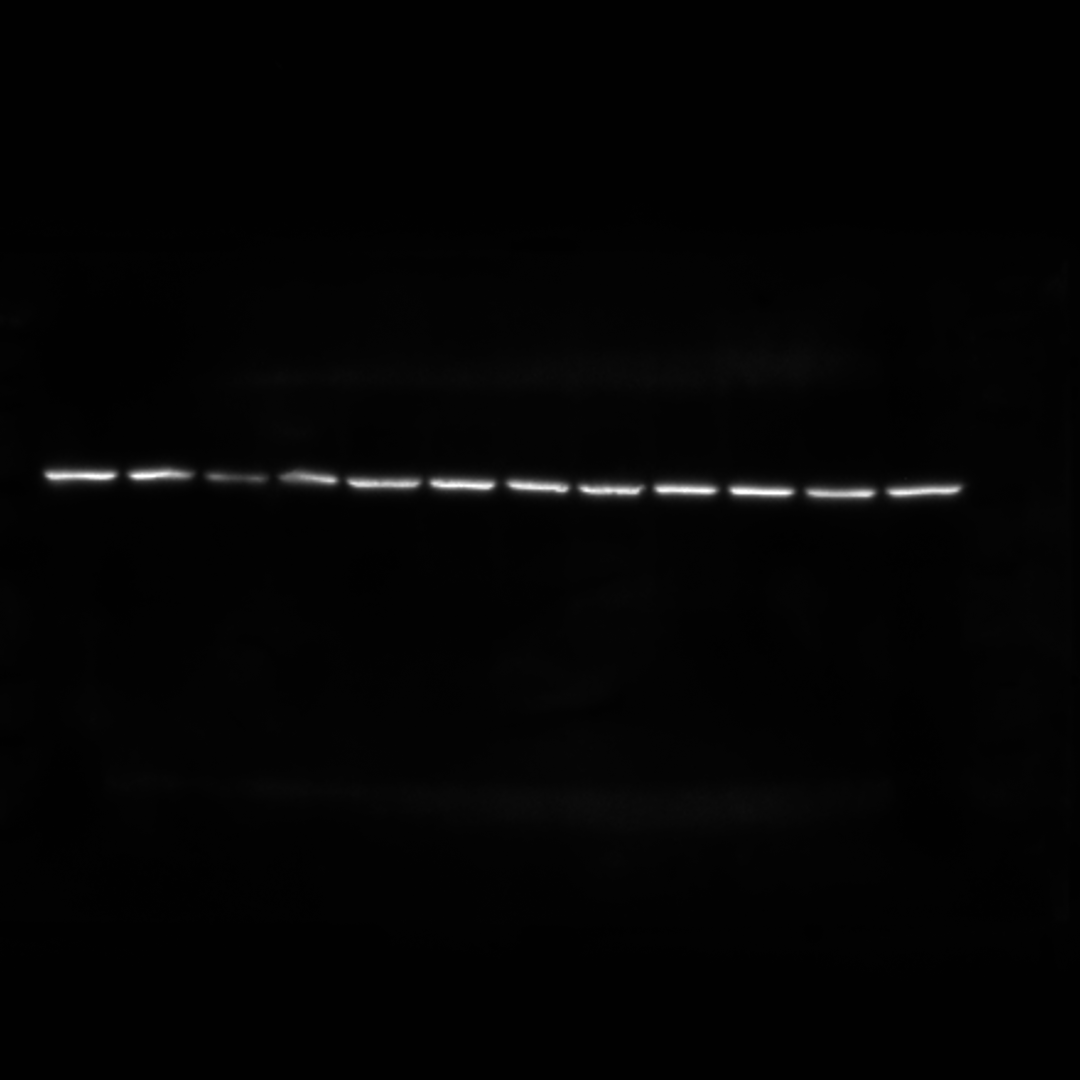

Supplement: Figure 4—figure supplement 1—source data 1. [file elife-99650-fig4-figsupp1-data1.zip › Figure 4 - figure supplement 1 - Source Data 1/Figure4_figuresupplement1B_Tubulin_raw.Tif]

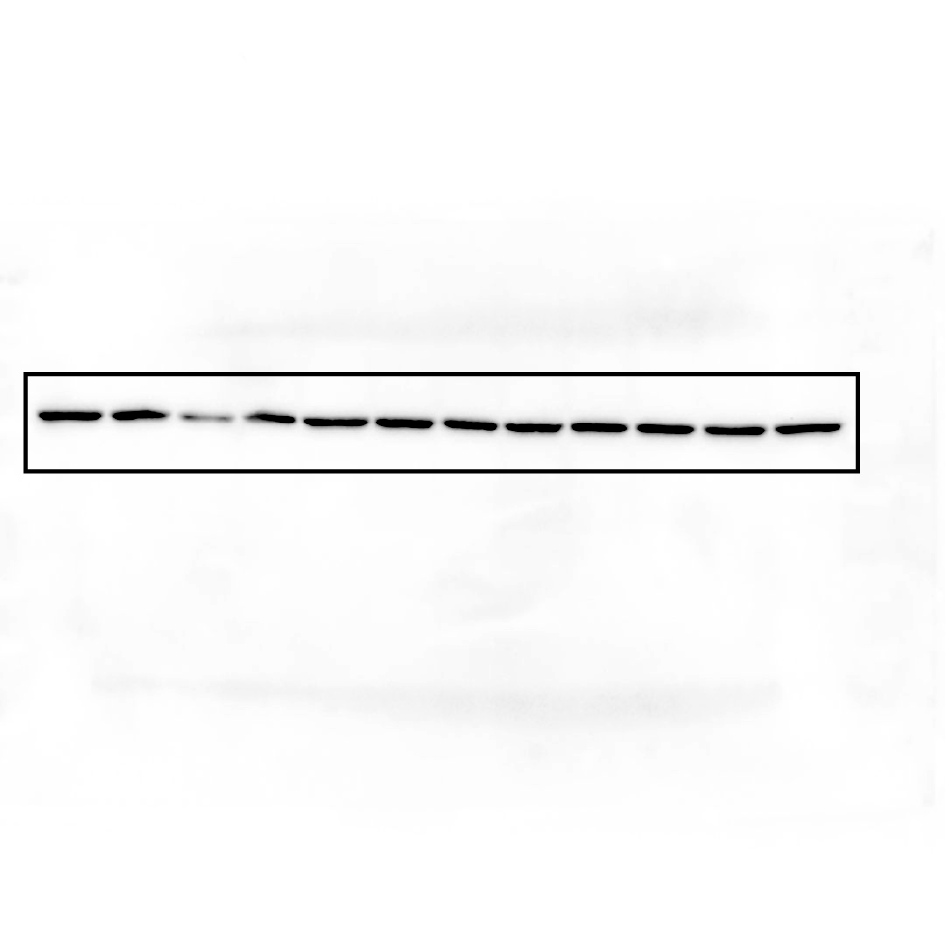

Supplement: Figure 4—figure supplement 1—source data 2. [file elife-99650-fig4-figsupp1-data2.zip › Figure 4 - figure supplement 1 - Source Data 2/Figure4_figuresupplement1B_Tubulin_annotated.tif]

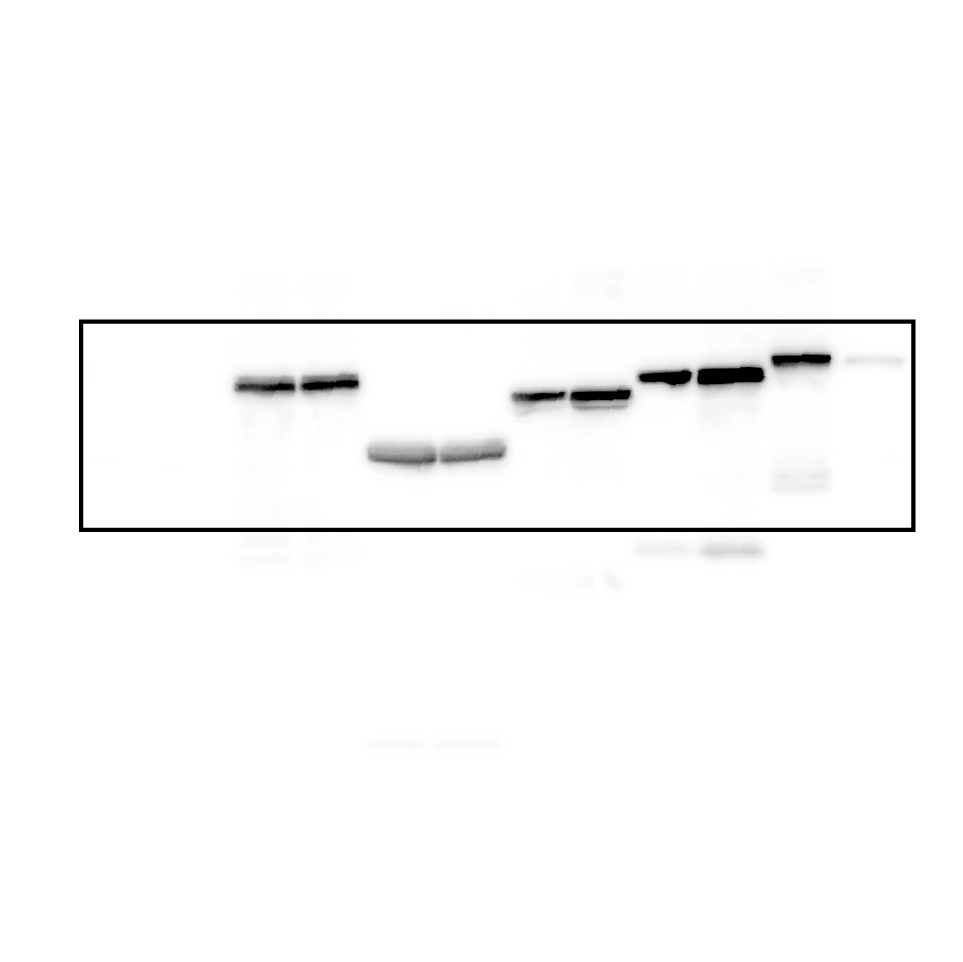

Supplement: Figure 4—figure supplement 1—source data 2. [file elife-99650-fig4-figsupp1-data2.zip › Figure 4 - figure supplement 1 - Source Data 2/Figure4_figuresupplement1B_FLAG_annotated.tif]

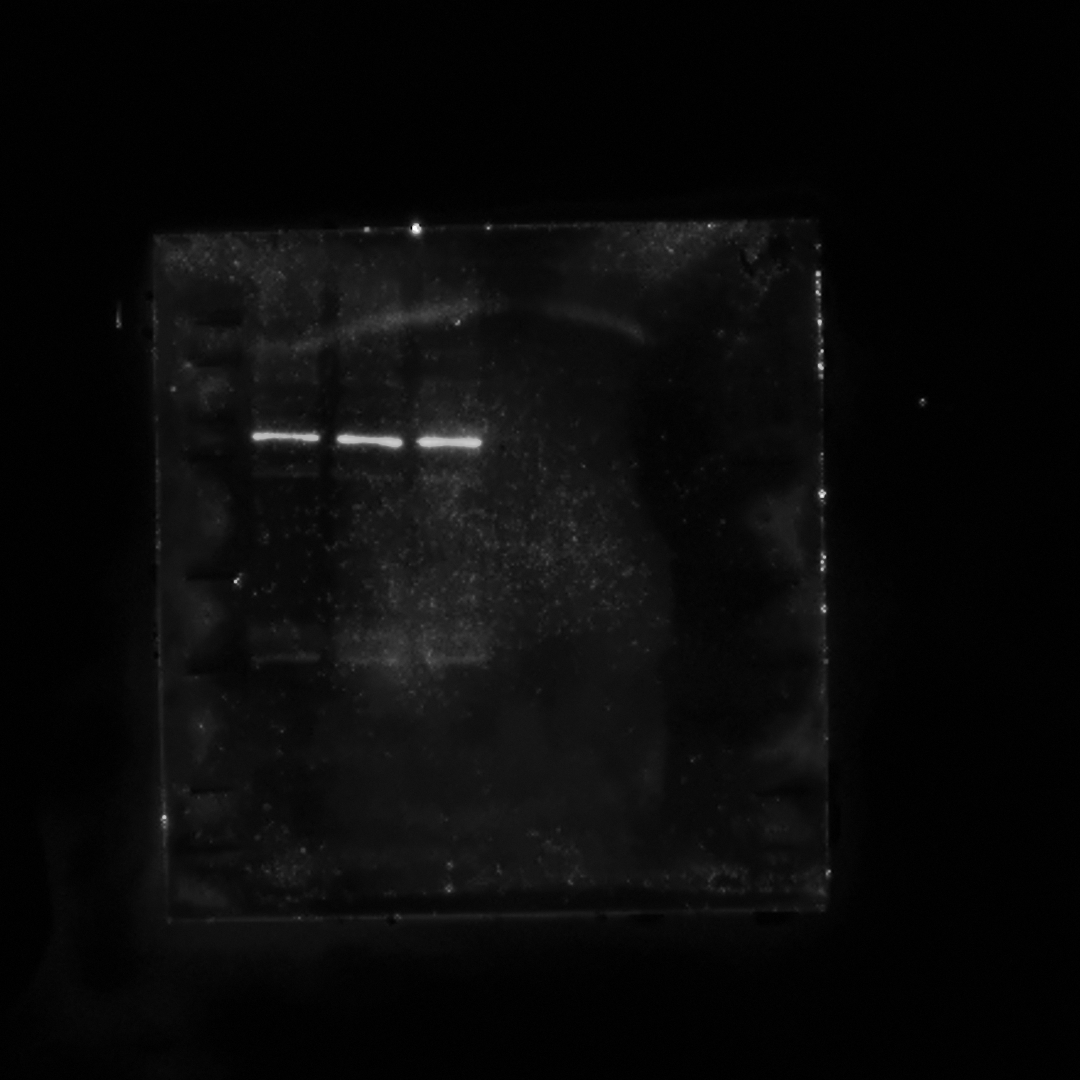

Supplement: Figure 5—source data 1. [file elife-99650-fig5-data1.zip › Figure 5 - Source Data 1/Figure5B_V5_raw.Tif]

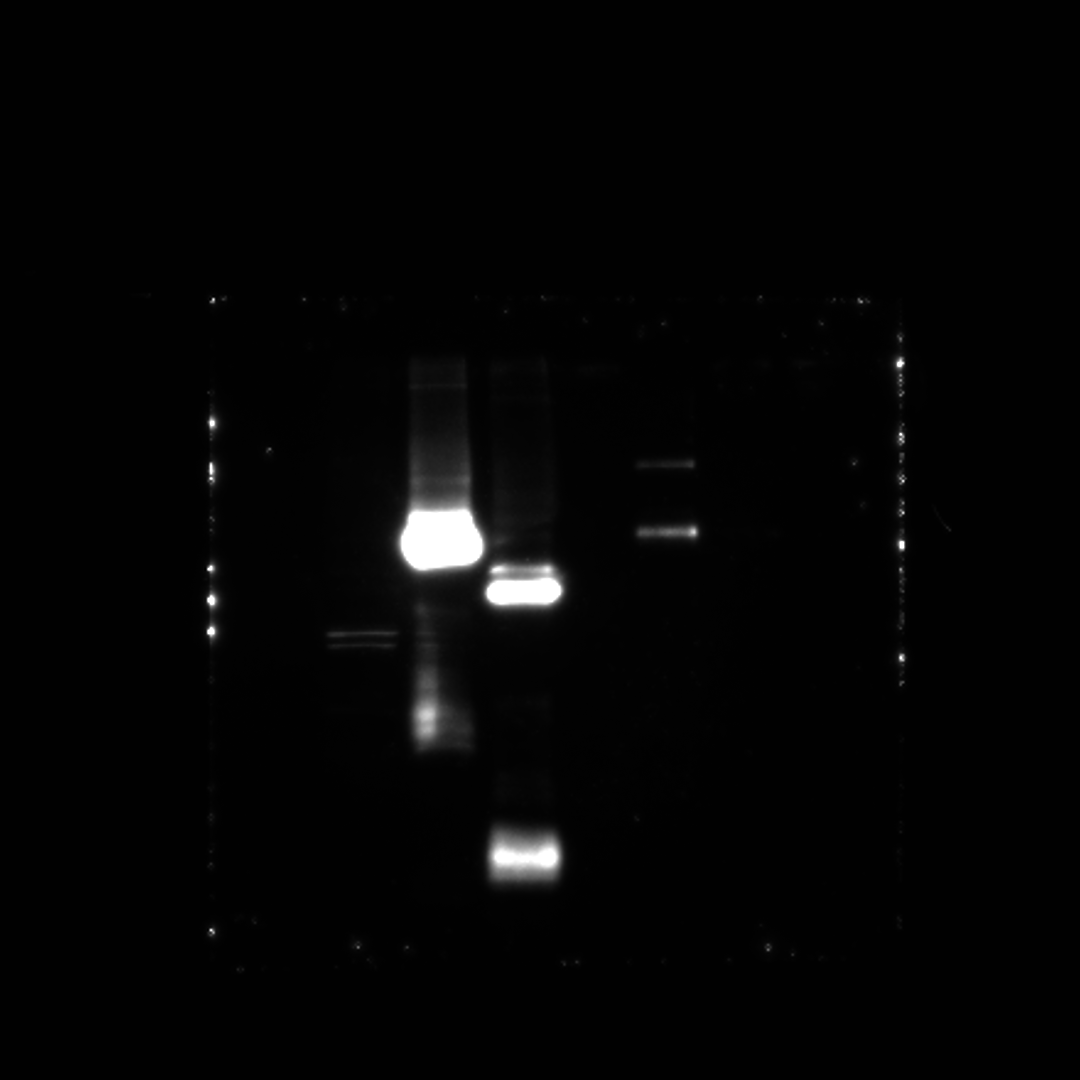

Supplement: Figure 5—source data 1. [file elife-99650-fig5-data1.zip › Figure 5 - Source Data 1/Figure5B_FLAG_raw.Tif]

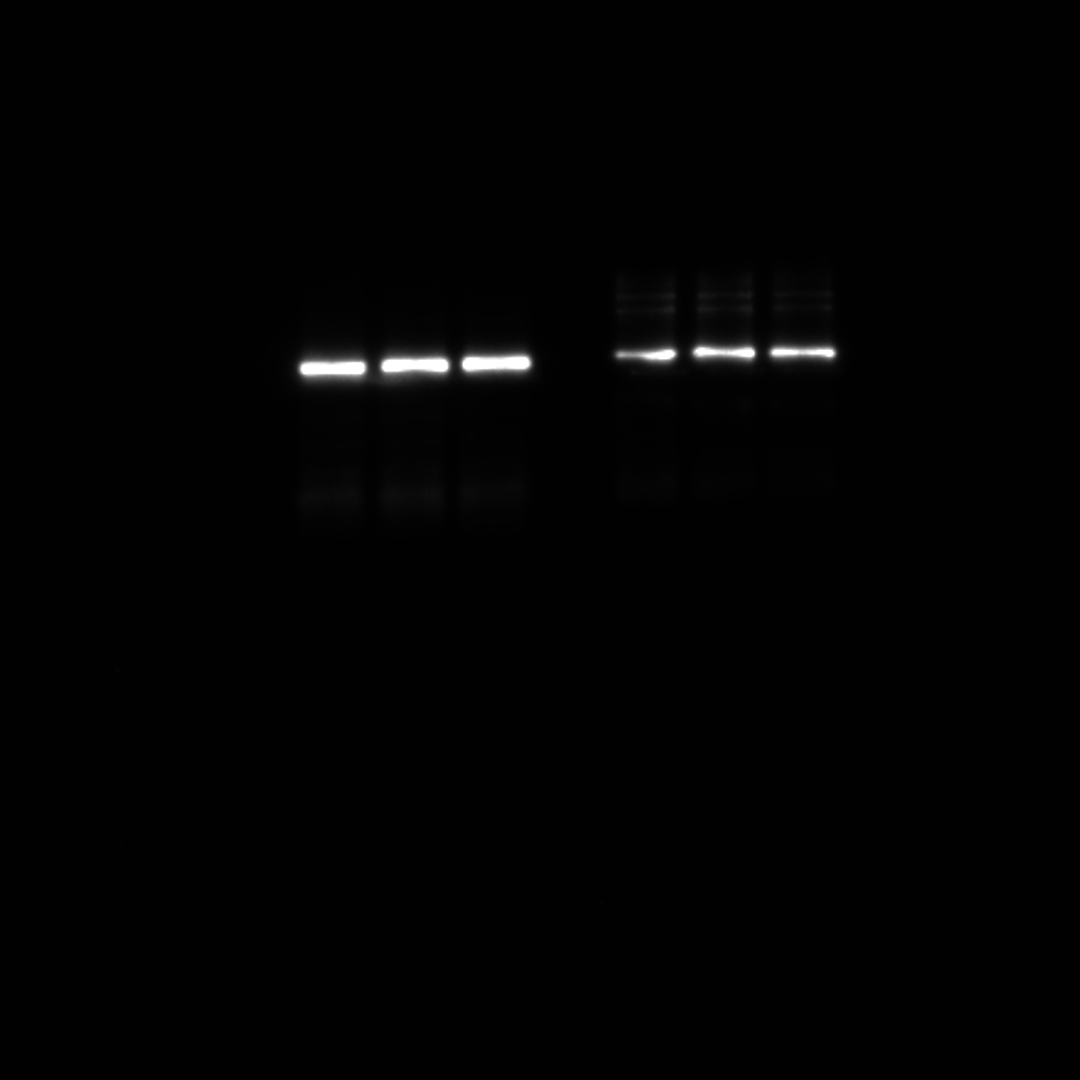

Supplement: Figure 5—source data 1. [file elife-99650-fig5-data1.zip › Figure 5 - Source Data 1/Figure5E_FLAG_raw.Tif]

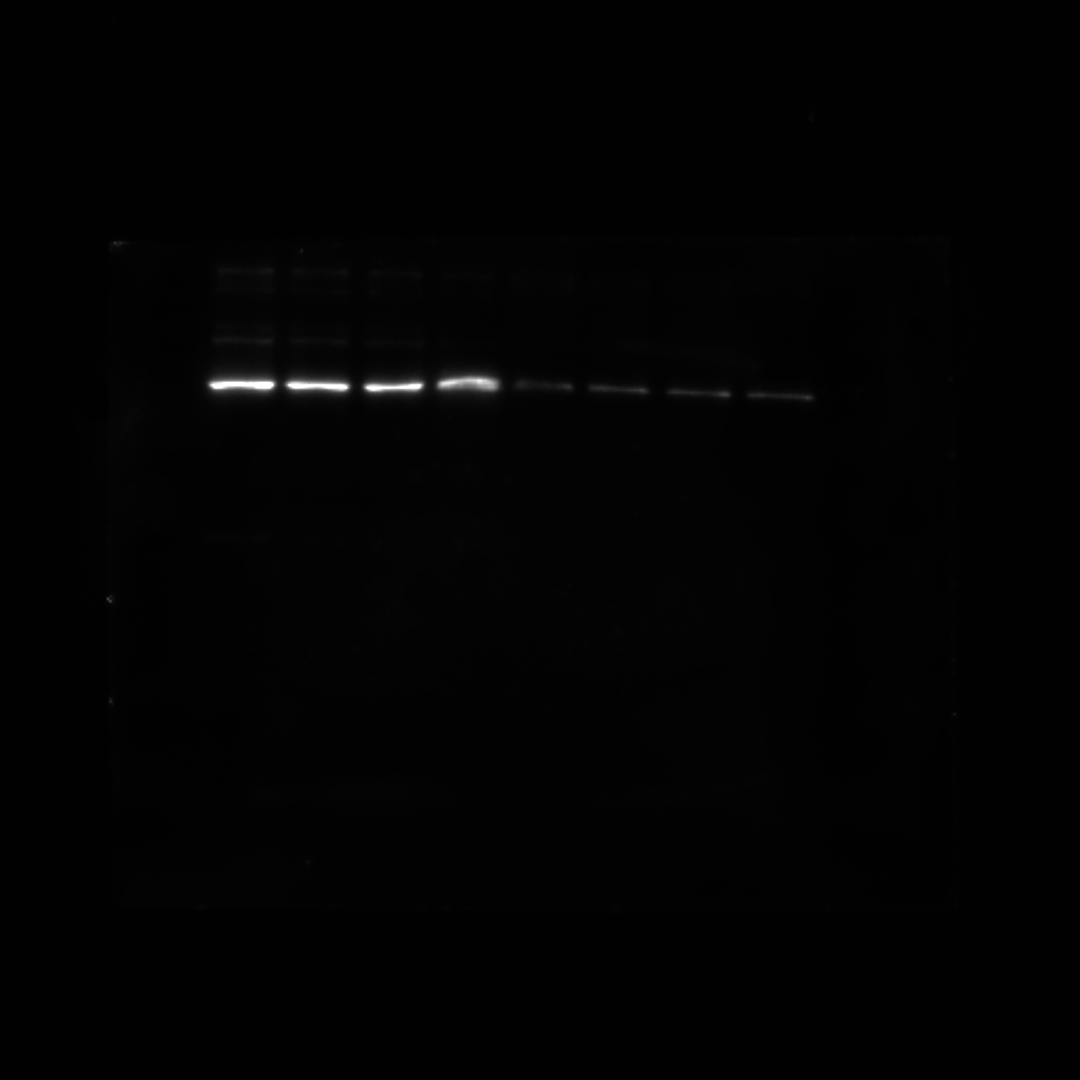

Supplement: Figure 5—source data 1. [file elife-99650-fig5-data1.zip › Figure 5 - Source Data 1/Figure5E_V5_raw.Tif]

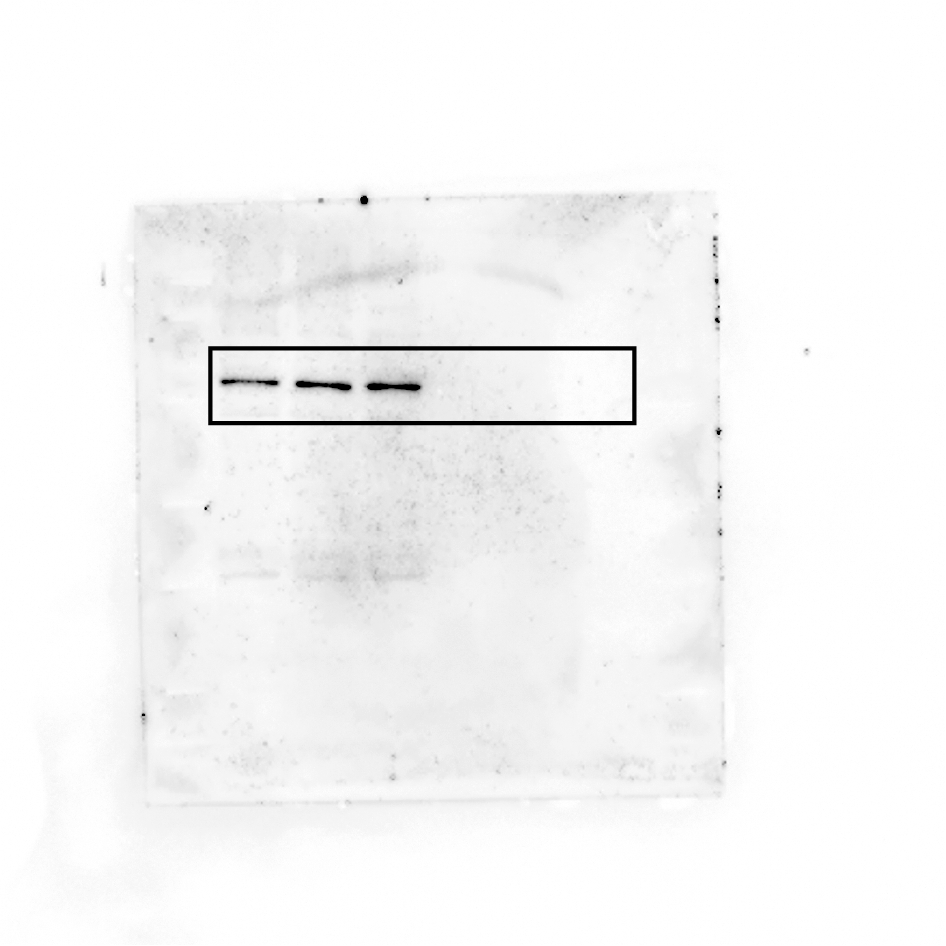

Supplement: Figure 5—source data 2. [file elife-99650-fig5-data2.zip › Figure 5 - Source Data 2/Figure5B_V5_annotated.tif]

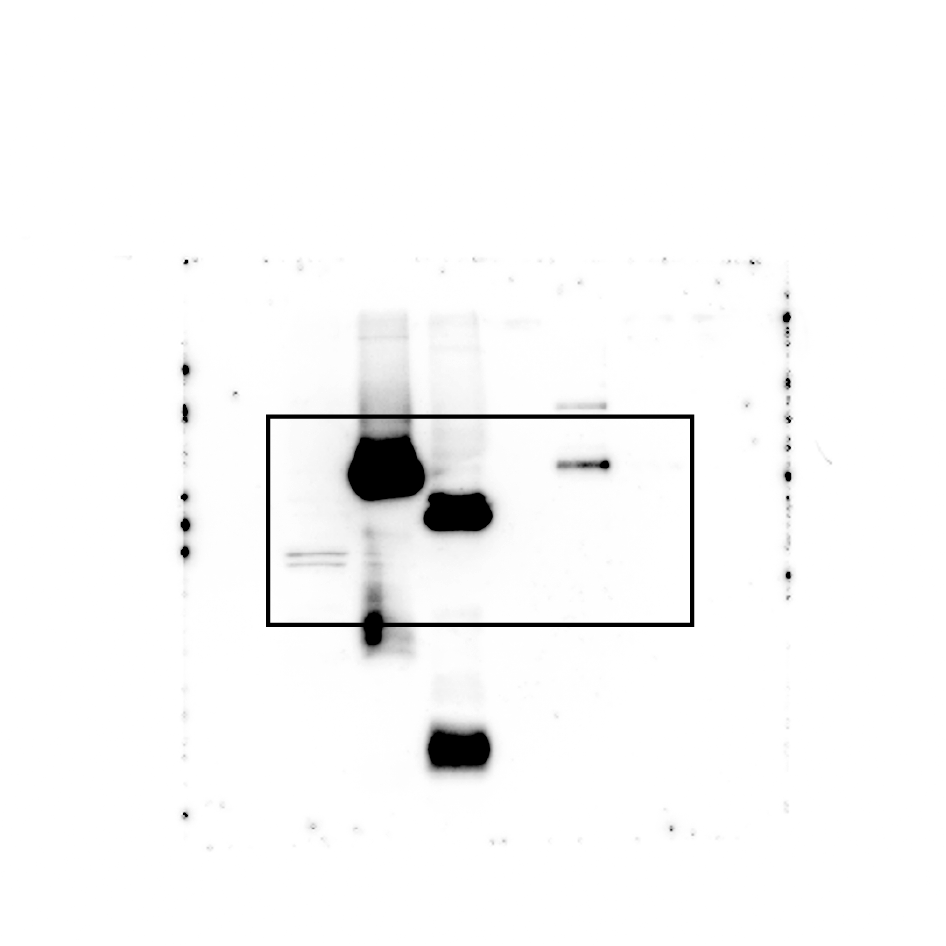

Supplement: Figure 5—source data 2. [file elife-99650-fig5-data2.zip › Figure 5 - Source Data 2/Figure5B_FLAG_annotated.tif]

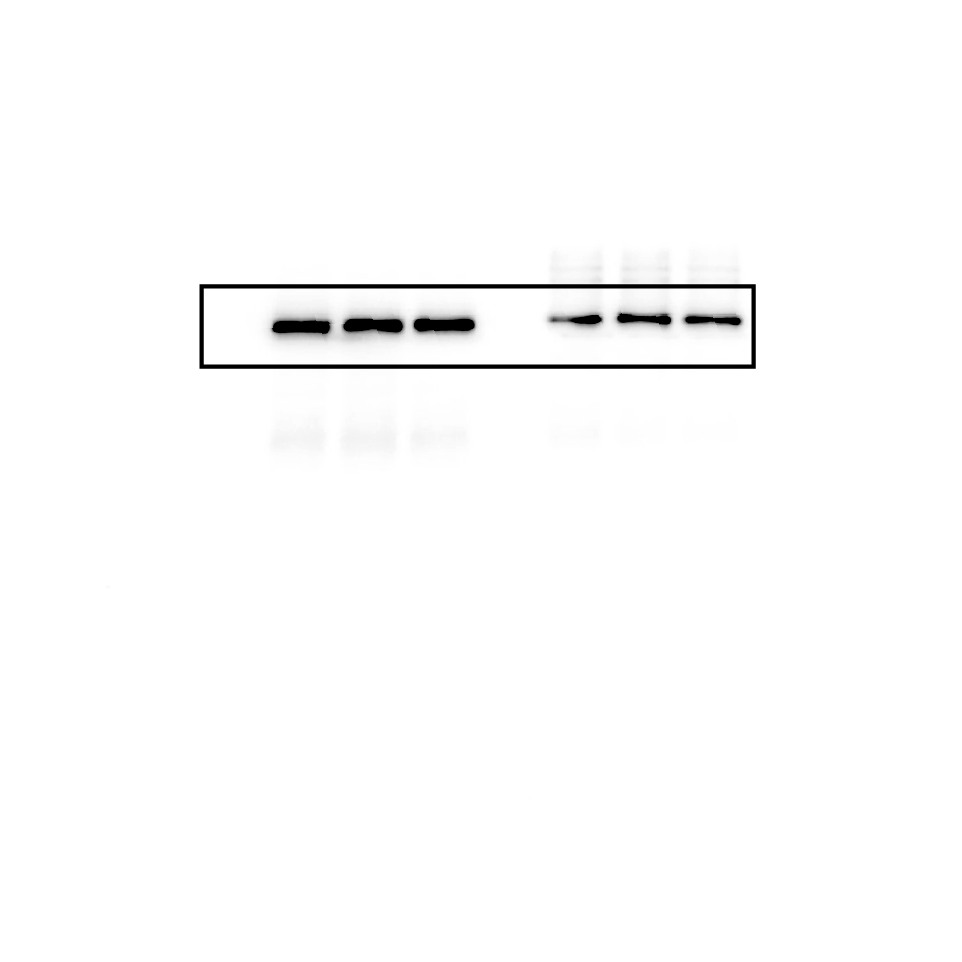

Supplement: Figure 5—source data 2. [file elife-99650-fig5-data2.zip › Figure 5 - Source Data 2/Figure5E_FLAG_annotated.tif]

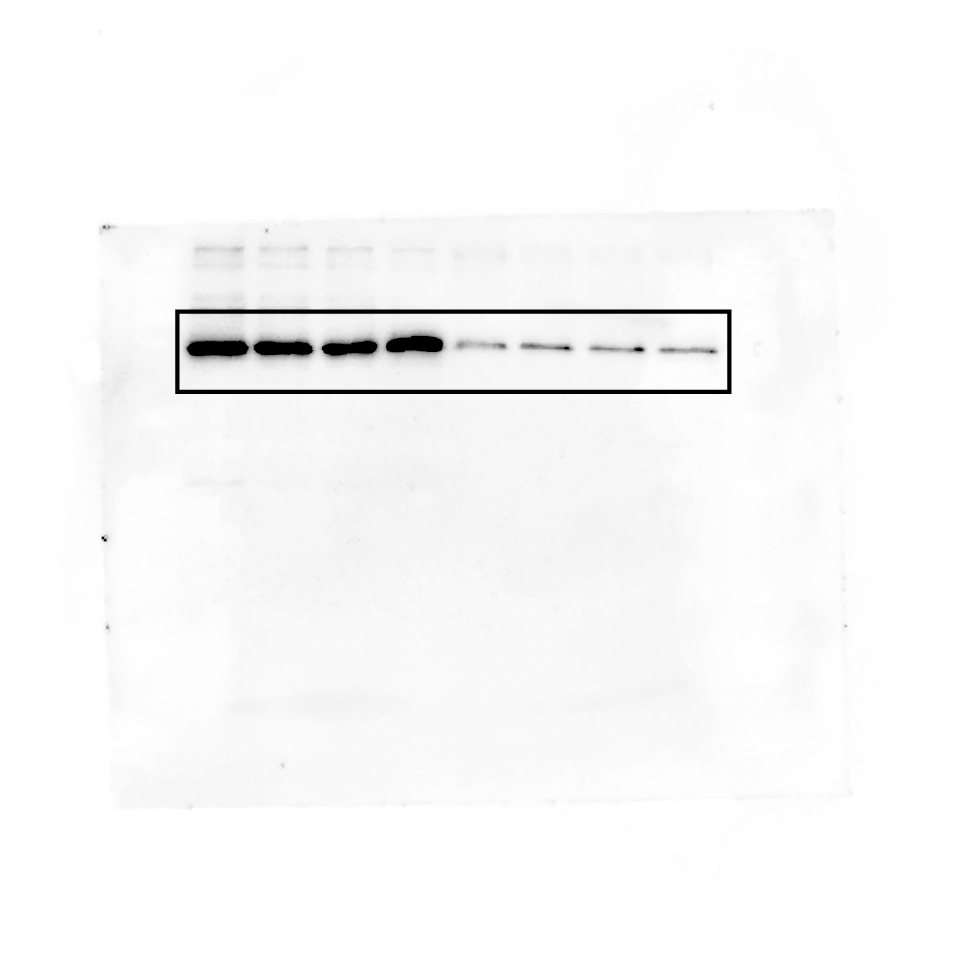

Supplement: Figure 5—source data 2. [file elife-99650-fig5-data2.zip › Figure 5 - Source Data 2/Figure5E_V5_annotated.tif]
